# Genetic disruption of *Plasmodium falciparum* Merozoite surface antigen 180 (PfMSA180) suggests an essential role during parasite egress from erythrocytes

**DOI:** 10.1038/s41598-021-98707-0

**Published:** 2021-09-28

**Authors:** Vanndita Bahl, Kritika Chaddha, Syed Yusuf Mian, Anthony A. Holder, Ellen Knuepfer, Deepak Gaur

**Affiliations:** 1 Laboratory of Malaria and Vaccine Research, School of Biotechnology, Jawaharlal Nehru University, New Delhi, 110067 India; 2 Malaria Parasitology Laboratory, The Francis Crick Institute, 1 Midland Road, London, NW1 1AT UK; 3 The Royal Veterinary College, Hawkshead Lane, North Mymms, Hatfield, AL9 7TA Hertfordshire UK

**Keywords:** Biotechnology, Molecular biology

## Abstract

*Plasmodium falciparum,* the parasite responsible for severe malaria, develops within erythrocytes. Merozoite invasion and subsequent egress of intraerythrocytic parasites are essential for this erythrocytic cycle, parasite survival and pathogenesis. In the present study, we report the essential role of a novel protein, *P. falciparum* Merozoite Surface Antigen 180 (PfMSA180), which is conserved across *Plasmodium* species and recently shown to be associated with the *P. vivax* merozoite surface. Here, we studied MSA180 expression, processing, localization and function in *P. falciparum* blood stages. Initially we examined its role in invasion, a process mediated by multiple ligand-receptor interactions and an attractive step for targeting with inhibitory antibodies through the development of a malaria vaccine. Using antibodies specific for different regions of PfMSA180, together with a parasite containing a conditional *pfmsa180*-gene knockout generated using CRISPR/Cas9 and DiCre recombinase technology, we demonstrate that this protein is unlikely to play a crucial role in erythrocyte invasion. However, deletion of the *pfmsa180* gene resulted in a severe egress defect, preventing schizont rupture and blocking the erythrocytic cycle. Our study highlights an essential role of PfMSA180 in parasite egress, which could be targeted through the development of a novel malaria intervention strategy.

## Introduction

Malaria remains a major vector-borne infectious disease afflicting underdeveloped countries in the tropics. Despite a significant recent reduction in malaria-associated global mortality, this disease still accounts for ~ 200 million clinical cases and half a million deaths annually. The primary impact is on children below the age of 5 years and pregnant women^1,2^. Six *Plasmodium* species cause malaria in humans: *P. falciparum*, *P. vivax*, *P. knowlesi* (a zoonotic infection), *P. malariae*, *P. ovale curtisi* and *P. ovale wallikeri*^3^, but *P. falciparum* is responsible for the most lethal form of the disease and causes more than 90% of all malaria-related mortality and morbidity. *P. falciparum* remains a significant global health problem due in part to the development of antimalarial resistance by the parasite and the emergence of insecticide-resistant mosquitoes^4^. The life cycle of these parasites is complex and has two obligate alternate hosts: the female *Anopheles* mosquito vector and the human host. The asexual blood stage in humans is responsible for all clinical symptoms and pathology associated with the disease, and generates gametocytes, the first sexual stage necessary for transmission to mosquitoes. Within the intraerythrocytic cycle, the parasite multiplies by schizogony, producing merozoites, which egress from the infected erythrocyte and invade new uninfected erythrocytes. Both egress and invasion are highly regulated processes, vital for malaria pathogenesis^5,6^. The identification and study of merozoite antigens involved in invasion and egress are essential imperatives for the design of effective antimalarial therapies^7^, however, many are uncharacterised and a substantial fraction of the *P. falciparum* genome lacks functional annotation^8^.

More than 50% of the parasite genes encode hypothetical proteins with little evidence of homologs in other organisms^9,10^. Most of the parasite proteins discovered to have a role in erythrocyte invasion are encoded by genes with a late schizont-stage transcriptional expression profile and the presence of a signal peptide and C-terminal transmembrane domain similar to known invasion ligands^11–13^. Bioinformatic analysis of *P. falciparum* transcriptome profiles led to the identification of *P. falciparum* Merozoite Surface Antigen 180 (MSA180; GeneID: PF3D7_1014100)^11–13^, a homolog of *P. vivax* MSA180, a protein that was reported to be expressed in schizont stages and located on the merozoite surface^14^. In a recent study Nagaoka et al.^15^ proposed that antibodies against PfMSA180 inhibit merozoite invasion in vitro and are involved in protection against malaria, based on an analysis with human serum samples from Thailand. The authors also proposed that the C-terminal region of PfMSA180 binds to the erythrocyte surface protein, CD47 (integrin associated protein)^15^. In addition, a piggybac insertional mutagenesis study identified the *pfmsa180* gene to be essential^8^. Another study by Tan et al*.*^16^ published earlier this year whilst this manuscript was under review identified MSA180 as a critical co-factor for SERA6, a parasitophorous vacuole (PV)-localized cysteine protease involved in dismantling of the erythrocyte cytoskeleton during merozoite egress. Both MSA180 and SERA6 are substrates of a subtilisin-like protease called SUB1^16^, which is secreted from exonemes into the PV at the start of the proteolytic cascade culminating in the sequential rupture of the PV membrane (PVM) and the erythrocyte plasma membrane.

Here, we have examined in more detail the functional importance of PfMSA180 during the asexual blood stage of the parasite life cycle. We raised polyclonal antibodies against four recombinant proteins representing the entire PfMSA180 to analyse the protein, and used CRISPR/Cas9 technology^17^ to generate parasites allowing DiCre-mediated inducible knockout of the gene to study the effect on parasite biology^18^. We find that PfMSA180 co-localises in part with merozoite surface protein 1 (MSP-1) and is processed in mature schizonts. However, PfMSA180-specific antibodies did not inhibit erythrocyte invasion. The knockout of *pfmsa180* blocked parasite egress from the erythrocyte, indicating that the protein’s function precedes a role in invasion, corroborated by findings in Tan et al.^16^. MSA180 is conserved across the *Plasmodium* genus and thus may serve as an essential protein involved in egress in all species, lending itself as a promising target for the design of parasite egress inhibitors and new therapeutic interventions.

## Results

### Bioinformatic identification of PfMSA180 in the asexual erythrocytic cycle of *P. falciparum*

We first identified and selected PfMSA180 based on the similarity of its gene expression profile to that of well-characterised merozoite invasion ligands of the Erythrocyte Binding Antigen (EBA) and Rhoptry protein Homolog (RH) families, during late schizogony. Transcriptome analyses had classified PfMSA180 as part of a cluster of invasion-related proteins, which also includes major invasion ligands: EBA-175 and RH5^11–13^. PfMSA180 transcription is maximum late in the asexual erythrocytic cycle of various laboratory-adapted parasite lines of distinct geographic origin, including 3D7 (Africa), HB3 (Honduras, Central America) and Dd2 (IndoChina) (http://plasmoDB.org/plasmo/app/record/gene/PF3D7_1014100). The MSA180 homolog in *P. vivax* has been described recently^14^, and others can be identified in *Plasmodium* species infecting rodents and non-human primates. The protein is predicted to possess an N-terminal signal peptide, indicating that it is exported and suggestive of a surface localization and a potential role in erythrocyte invasion. PfMSA180 has a number of predicted Subtilisin-like protease 1 (SUB1) processing sites^19^ and numerous fragments of the protein have been identified in parasite extracts. PfSUB1 is a *Plasmodium* serine protease that plays a crucial role in parasite egress^20^ and invasion^21^. A multiple sequence alignment of MSA180 from five human malaria-causing species show that the amino acid sequence is conserved primarily at the N- and C-terminus of the protein (Supplementary Fig. 1).

### Production of PfMSA180 as a series of recombinant proteins

**Figure 1:**
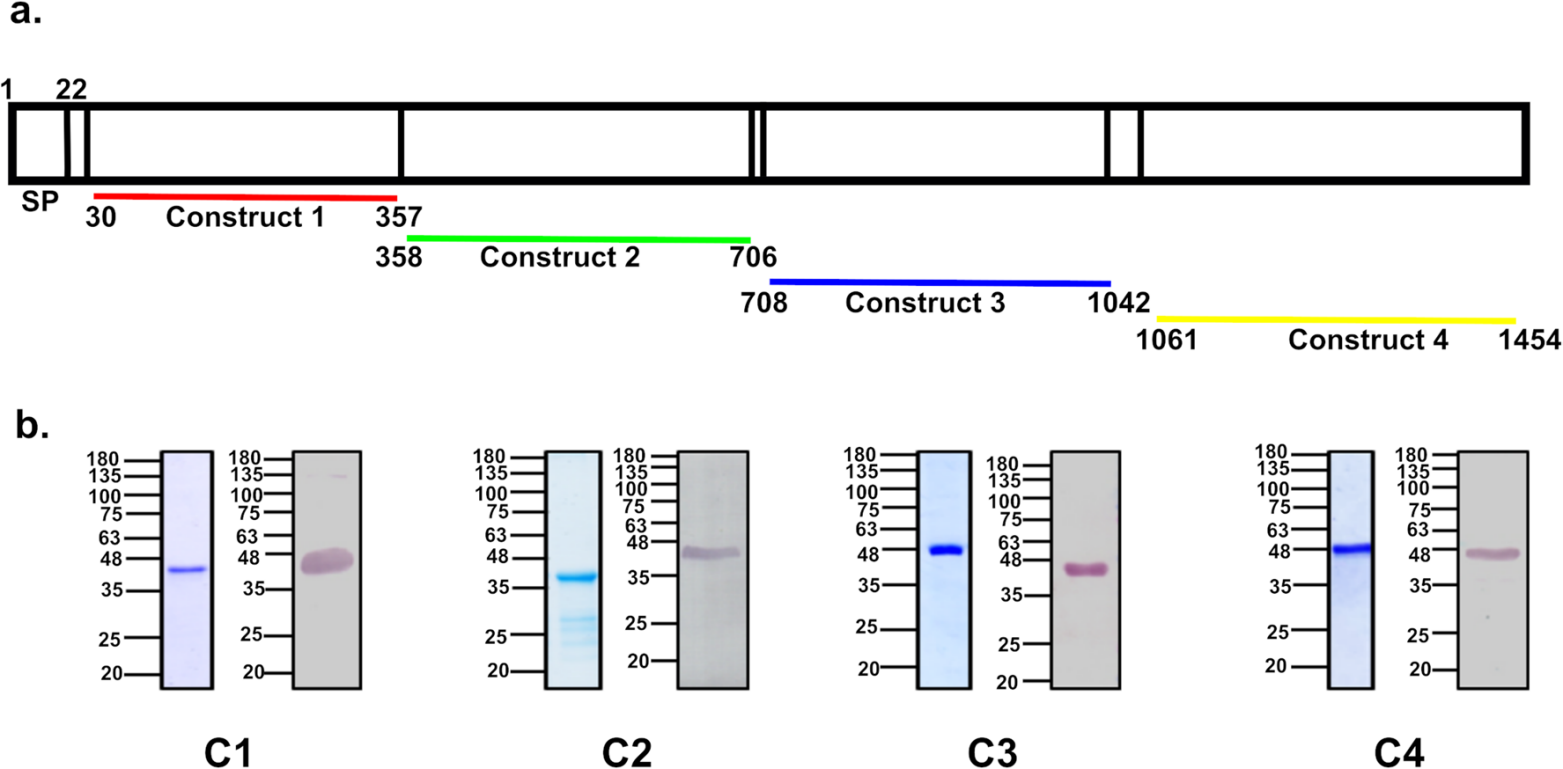
Expression of PfMSA180 as four recombinant proteins in *E. coli* and generation of specific antibodies. PfMSA180 is synthesized as a ~ 180 kDa protein in the parasite with a predicted N-terminal signal peptide (SP). The four recombinant proteins were expressed from plasmid constructs that together span the entire length of the protein. The constructs were based on the pET24b vector for expression in *E. coli* and the proteins were purified by Immobilised Metal Affinity Chromatography (IMAC) and size exclusion chromatography. (**a**) Schematic representation of different constructs designed for PfMSA180. The protein possesses a signal peptide (SP; residues 1–22). (**b**) Purified PfMSA180 recombinant proteins: Construct 1 (C1) spans the N-terminal region of the protein (residues N_30_-T_357_) and has a predicted molecular mass of 39 kDa, Construct 2 (C2; residues N_358_-N_706_) has a predicted molecular mass of 41.5 kDa, Construct 3 (C3; residues N_708_-D_1042_) has a predicted molecular mass of 40 kDa, and Construct 4 (C4) spans the C-terminal region of the protein (residues N_1061_-N_1454_) and has a predicted molecular mass of 47 kDa. The purified proteins were separated by SDS-PAGE and stained with Coomassie blue, as well as transferred and their identity and molecular mass confirmed by immunoblotting with anti-6XHis antibody. Full length gels and immunoblots are presented in Supplementary Fig. 8. The lower bands observed on Coomassie blue-stained gels of C2, C3 and C4 were not specifically identified by anti-His antibody, suggesting slight degradation of the protein during refolding and dialysis.

PfMSA180 has a predicted molecular mass of ~ 173 kDa and was recombinantly expressed in the form of four continuous fragments, spanning the entire protein (Fig. 1a). These constructs were designed such that they maintain the secondary structural motifs of the full -length protein, which was predicted to comprise around 42% α-helix, 15% β-strand and 40% disordered regions using the online protein structure prediction tool, Protein Homology/AnalogY Recognition Engine (Phyre2)^22^. The constructs were cloned in the pET24b vector and expressed in *E. coli* to produce recombinant proteins with a C-terminal hexa-histidine tag. The N-terminal C1 recombinant protein was soluble and purified directly from bacterial lysate using immobilized metal affinity chromatography (IMAC), while the other three proteins (C2, C3 and C4) were expressed in inclusion bodies and therefore purified under denaturing conditions using IMAC (Supplementary Fig. 2) followed by refolding in vitro*.* All four proteins were further purified using size exclusion chromatography (Fig. 1b).

### Antibodies raised against the recombinant proteins identify PfMSA180 in parasite lysates, colocalize with anti-MSP-1 antibodies in schizonts, but do not inhibit merozoite invasion

**Figure 2:**
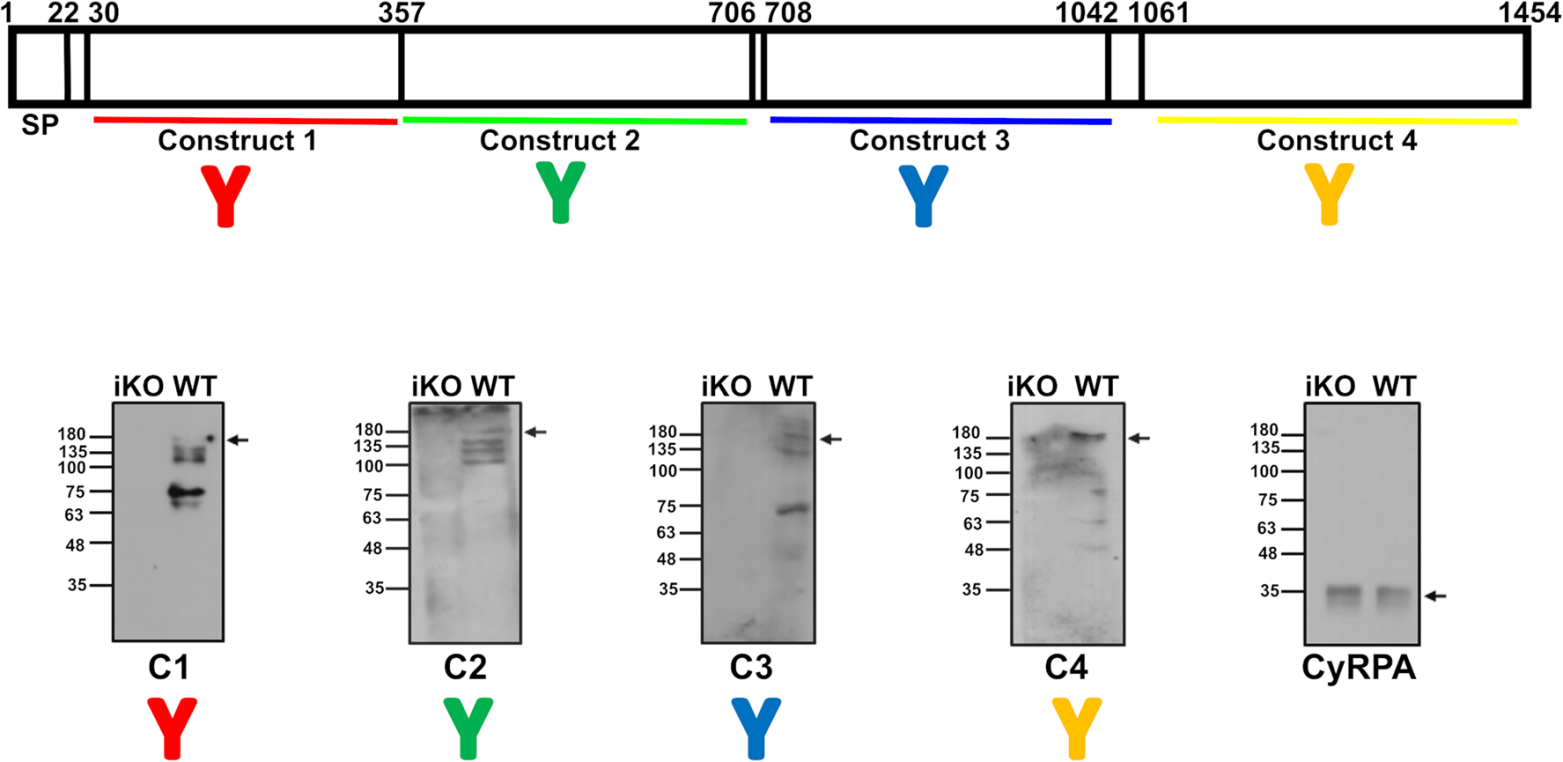
Immunoblotting with antibodies raised to PfMSA180-derived recombinant proteins detects the full-length PfMSA180 and displays processing of the protein in late schizont stages. Polyclonal antibodies raised in mice against the PfMSA180 recombinant proteins specifically detect the ~ 180 kDa protein in *P. falciparum* schizont lysates. WT- Wild type: schizont lysate prepared from control DMSO treated parasites, iKO- inducible knockout: schizont lysate prepared from rapamycin-treated parasites lacking MSA180 expression (see Fig. 5). Immunoblot analysis further detected additional polypeptides that may be fragments consistent with the protein undergoing proteolytic cleavage. Antibodies targeting N-terminal C1 recombinant protein identified the full-length protein (~ 180 kDa), and processed fragments of approximately 135 kDa, 100 kDa, 75 kDa and 63 kDa. C2-construct antibodies identified full-length protein (~ 180 kDa), and processed fragments of approximately 125 kDa and 96 kDa. C3-antibodies identified full-length protein (~ 180 kDa), and processed fragments of approximately 125 kDa and 75 kDa. C-terminal C4-antibodies identified full-length protein (~ 180 kDa), and processed fragments of approximately 75 kDa, 63 kDa and 48 kDa. Immunoblotting of the same parasite lysate preparation with anti-CyRPA antibodies on the same membrane was performed as loading control for late schizont stages. Full length immunoblots are shown in Supplementary Fig. 9.

Antibodies against the PfMSA180 recombinant proteins were generated in rabbits and mice as described previously^23^ (Supplementary Figs. 3 and 4) and specifically detected PfMSA180 in parasite extracts by immunoprecipitation (Supplementary Table 2) and immunoblotting (Fig. 2). Antibodies generated with all four recombinant proteins identified a ~ 180 kDa protein in *P. falciparum* 3D7 parasite lysate, corresponding to the full-length protein, and a number of smaller polypeptides that may represent processed fragments of the protein in schizonts of *P. falciparum*, as reported previously^15^ (Fig. 2). Based on our analysis the protein was predicted to possess three potential SUB1 protease cleavage sites as identified by the presence of a SUB1 conserved motif Ile/Leu/Val/Thr–Xaa–Gly/Ala-Paa-Xaa (with high prevalence of Glu/Asp/Ser/Thr) in which Xaa is any residue and Paa are polar amino acids^19^ (Supplementary Fig. 5). All four PfMSA180 antibodies identified the full-length protein (~ 180 kDa) as well as several processed protein fragments. The N-terminal C1 antibody identified bands of 135 kDa, ~ 100 kDa, 75 kDa and ~ 63 kDa; C2 antibodies identified bands of 135 kDa, ~ 125 kDa and ~ 100 kDa; C3 antibodies identified bands of ~ 125 kDa and 75 kDa; and C-terminal C4 antibodies identified bands of 75 kDa, 63 kDa and 48 kDa. Although we cannot rule out the possibility that some of these bands are fragments of MSA180 produced by degradation during lysate preparation, a number of bands correspond in size to the apparent molecular masses of polypeptides predicted from proposed SUB1 cleavage sites (Supplementary Fig. 5) and therefore are likely processing products. Immunoblots in Tan et al*.*^16^ using schizont lysates from a transgenic *MSA180-HAx3:loxP* parasite line and anti-HA antibodies identified full-length as well as processing bands corresponding to ~ 120–140 kDa, ~ 85 kDa and ~ 55 kDa fragments, which could correspond to bands ~ 135 kDa, ~ 125 kDa, ~ 75 kDa and ~ 48 kDa seen in this study lacking the triple HA-tag.

**Figure 3:**
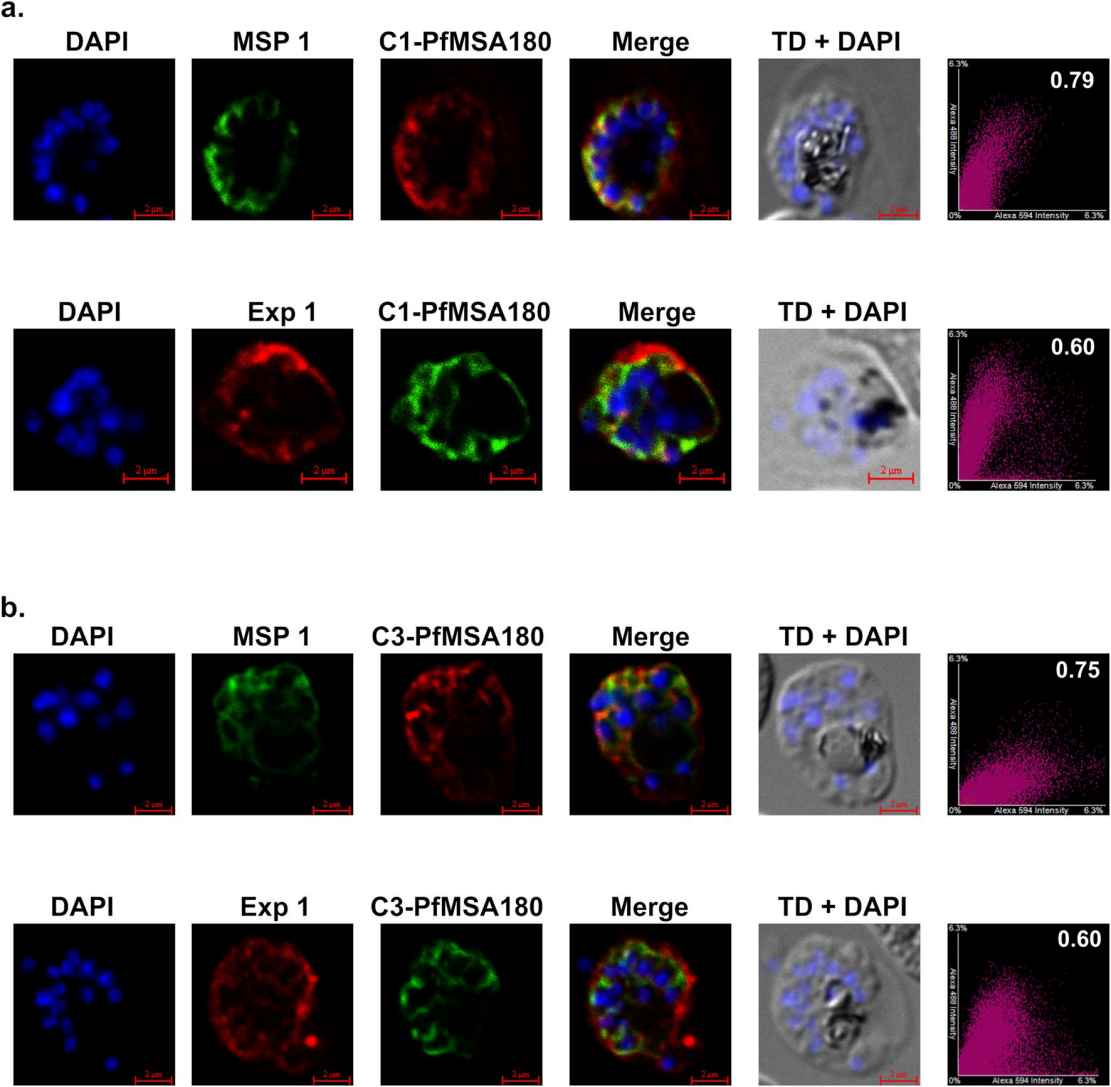
Immunofluorescence microscopy showing co-localisation of PfMSA180 with MSP-1 within mature schizonts. (**a**) Immunofluorescence detection of antibodies to C1 (N-terminus of PfMSA180) showing areas of co-localisation with MSP1 antibodies. Upper panel C1-PfMSA180 (Red), MSP1 (Green). Second panel C1-PfMSA180 (Green), Exp1 (Red). (**b**) Immunofluorescence detection of antibodies to C3 (C-terminus of PfMSA180) showing areas of co-localisation with MSP1 antibodies. Upper panel C3-PfMSA180 (Red), MSP1 (Green). Second panel C3-PfMSA180 (Green), Exp1 (Red). MSP1 is a merozoite surface protein and Exp1 (Exportin 1) is a parasitophorous vacuole membrane marker protein. The first column shows DAPI (4′,6-diamidino-2-phenylindole) DNA-stained schizonts, the second column shows the staining observed with marker antibodies, the third column displays PfMSA180 antibody staining, the fourth column is a merged image of the antibody staining, and the fifth column shows the transmitted image (TD) together with DAPI staining. The last column is the graph for antibody staining to represent Pearson’s correlation coefficient for co-localisation. The correlation coefficient is a measure of signal intensities of the two fluorophores in one image and the magnitude of the coefficient predicts the spatial relationship of the two fluorophores. A value close to zero indicates no correlation between the two fluorophores, while a value close to 1 indicates a positive correlation between the two fluorophores^42^. Five images were captured for each antibody combination and correlation coefficient values for colocalisation of anti-MSA180 (C1, C3) with anti-MSP1 ranged from 0.75–0.79, while the correlation coefficient of anti-MSA180 (C1, C3) with anti-Exp1 ranged from 0.56–0.60. One image for each antibody combination is displayed here.

Immunofluorescence microscopy with fixed cells and antibodies generated to the N-terminal (C1) and C-terminal (C3) recombinant proteins was used to investigate the subcellular location of PfMSA180. Although PfMSA180 possesses no predicted transmembrane domains or GPI-anchor, Nagaoka et al.^15^ have previously detected PfMSA180 tightly associated with the surface of merozoites, whereas Tan et al.^16^ have shown *MSA180-HAx3:loxP* to be predominantly soluble in the PV before forming foci partially co-localising with ankyrin upon egress. Here our PfMSA180-specific antibodies exhibited an approximate co-localisation with MSP1 specific antibodies^24^ in the parasitophorous vacuole (PV) of mature schizonts, suggesting that PfMSA180 is on or close to the merozoite surface in developing intracellular schizonts. Because of the suggested presence of PfMSA180 in a membrane fraction^15^, we therefore also examined co-localisation of PfMSA180 with Exportin-1 (Exp1), a protein located in the parasitophorous vacuolar membrane (PVM)^25^. PfMSA180-specific antibodies co-localized relatively poorly with Exp1-specific antibodies, consistent with a better co-localisation within the PV space with the merozoite surface rather than the PVM in developing schizonts (Fig. 3).

**Figure 4:**
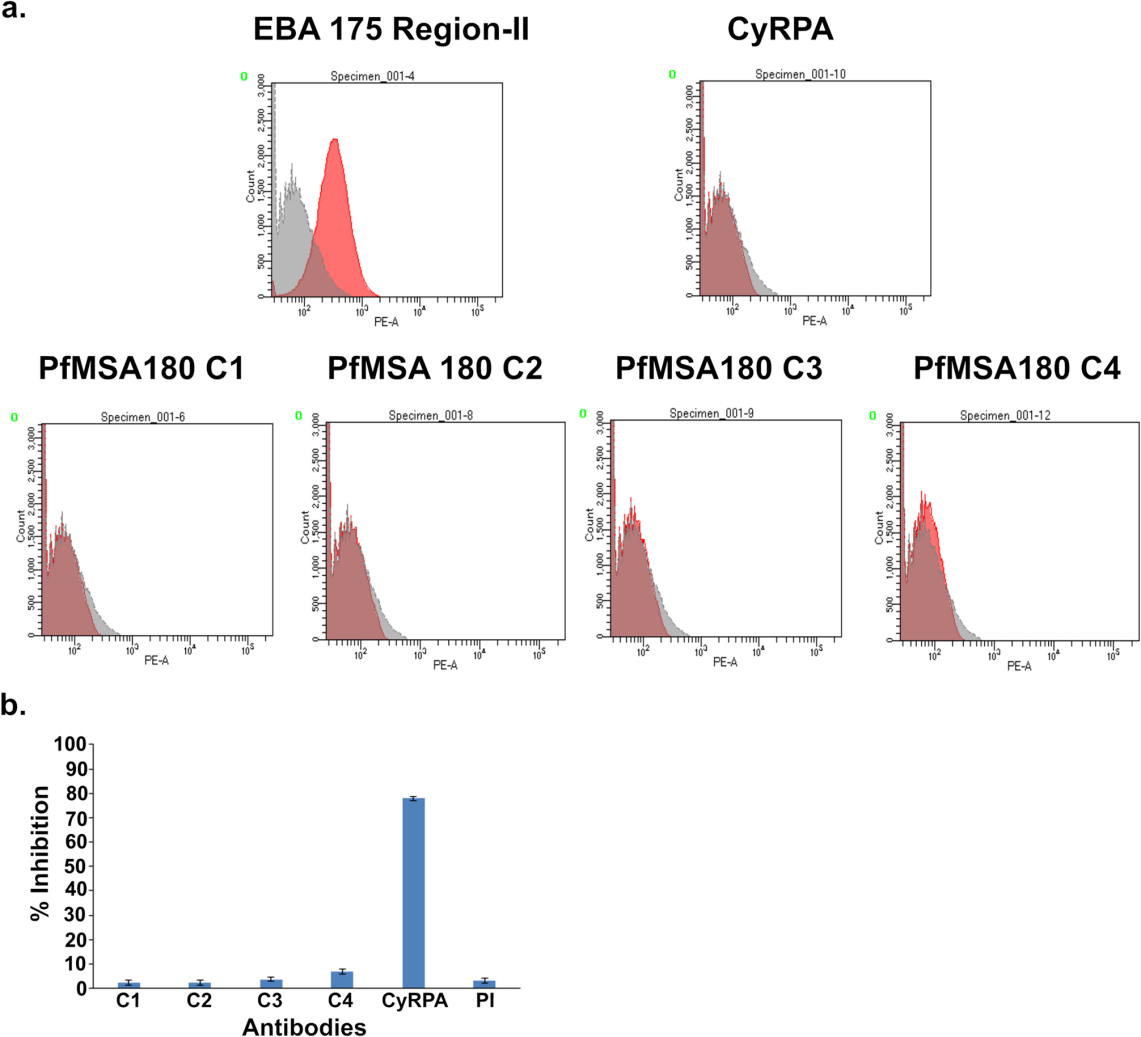
PfMSA180-derived recombinant proteins do not bind erythrocytes and MSA180-specific antibodies do not inhibit invasion of erythrocytes by *P. falciparum *in vitro*.* (**a**) Recombinant PfMSA180 proteins fail to bind erythrocytes in a FACS-based binding assay. 10 µg of each recombinant protein was used. EBA175 region II and CyRPA were used as positive and negative controls. One of two experiments is shown. (**b**) Antibodies raise against PfMSA180 recombinant proteins failed to inhibit erythrocyte invasion in an in vitro Growth Inhibition Assay (GIA). 10 mg/ml of purified rabbit antibody were used and each exhibited < 5% inhibition. Rabbit antibodies targeting CyRPA were used as a positive control. Three independent assays were performed in duplicate. The error bars show the standard error of the mean.

We also investigated the erythrocyte binding activity of the recombinant proteins using a FACS-based assay (described in Methods section). However, none of the four recombinant PfMSA180 proteins had significant erythrocyte binding activity, while, the Erythrocyte binding antigen 175 region II (EBA-175RII), used as a positive control, exhibited a strong erythrocyte binding activity^26^ (Fig. 4a).

Next, we studied the ability of the PfMSA180-specific antibodies to inhibit merozoite invasion in an in vitro growth inhibition assay (GIA). Immunoglobulin G (total IgG) was purified from both pre-immune (PI) and day 70 rabbit sera (after immunization), and tested at 10 mg/ml in the assay. The purified IgGs against the four PfMSA180 recombinant proteins exhibited negligible invasion inhibition, as measured relative to the pre-immune IgG control. In contrast, CyRPA-specific antibodies, used as a positive control, exhibited potent inhibition of invasion, as reported previously^27^ (Fig. 4b).

### Generation of a parasite line for inducible knockout of the *msa180* gene to study its role in the asexual blood stage of the parasite life cycle

**Figure 5:**
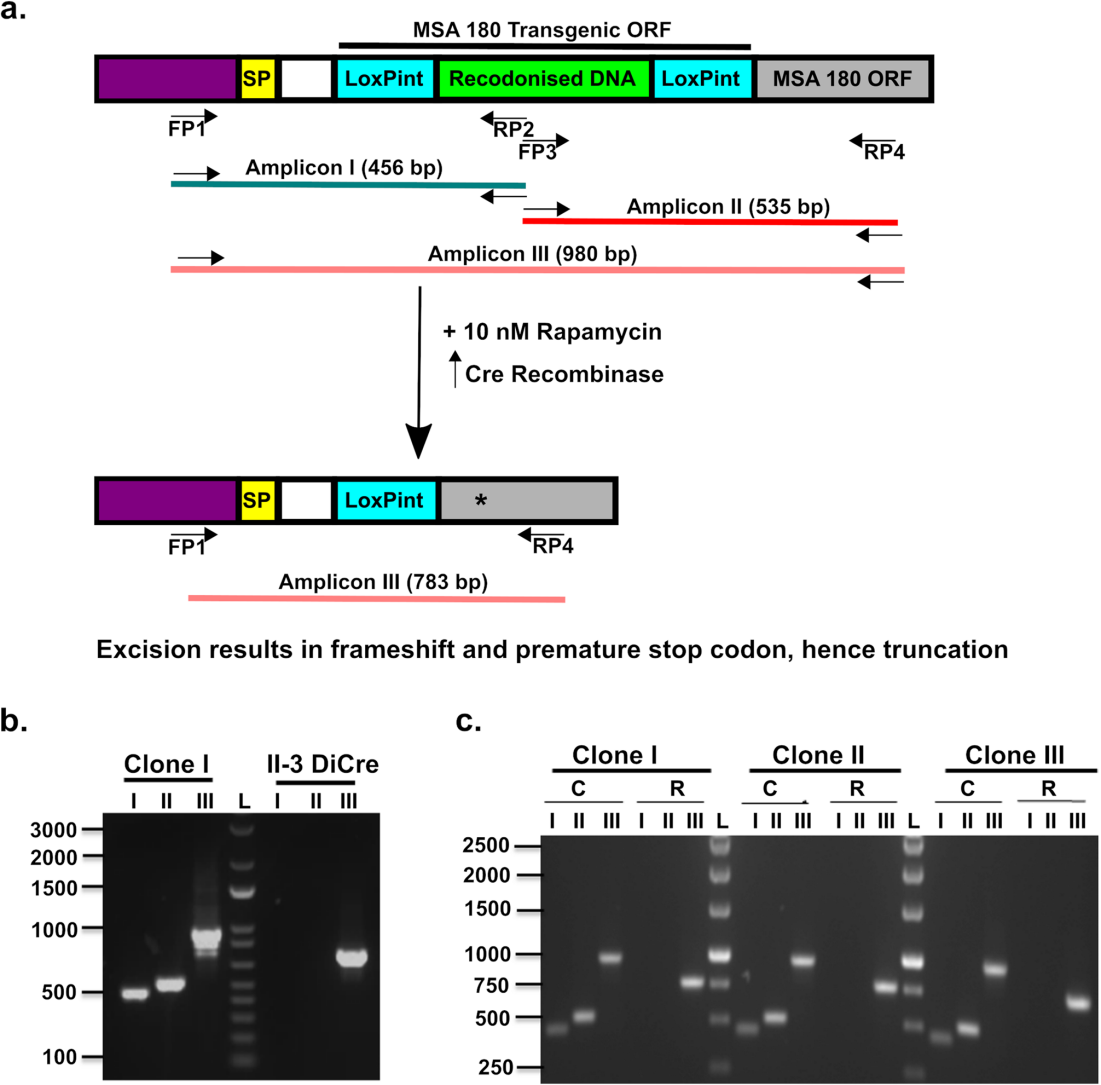
Generation of parasite lines to perform inducible knockout of the *pfmsa180* gene. (**a**) Schematic diagram of the expected amplicons with different primer pairs. The recondonised DNA between the two LoxPint sites was used to differentiate the transfected (PfMSA180) and wild type (II-3 DiCre) parasite lines. SP- Signal peptide, FP- Forward Primer, RP- Reverse primer, *premature stop codon after successful recombination, LoxPint- LoxP sites in *sera2* intron. Predicted amplicon I (456 bp) was amplified with primer pairs 1/2, Amplicon II (535 bp) was amplified with primer pairs 3/4, Amplicon III was amplified with primer pairs 1/4 and the size depended on rapamycin treatment (without rapamycin/DMSO treated, 980 bp; after rapamycin treatment, 783 bp). The transgenic ORF is comprised of two LoxPint sites inserted at the 5’ end of the gene; the remaining *msa180* ORF is depicted by grey box. Not to scale. (**b**) PCR to confirm transfection and insertion of repair sequence. Lane1- Amplicon I (Primers 1/2), Lane 2- Amplicon II (Primer 3/4), Lane 3- Amplicon III (Primers 1/4) from *pfmsa180* inducible knockout clone (980 bp) and from wildtype II-3 DiCre parasites (774 bp). PCR results shown for one of three *pfmsa180* inducible knockout parasite clones. PfMSA180 transgenic line – the presence of a band in Lane 1 and 2 of MSA180 confirms integration of the repair sequence containing two LoxPint sites into the parasite genome. Lane 3 confirms the presence of unmodified *msa180* sequence in wildtype parasites. (**c**) PCR analysis of DNA excision after rapamycin treatment for all three inducible *msa180* knockout clones. Early ring-stage parasites were treated with 10 nM rapamycin and the growth medium was changed after 24 h without adding further rapamycin. L- DNA Ladder, Lane1-Amplicon I (Primers 1/2), Lane 2-Amplicon II (Primer 3/4), Lane 3-Amplicon III (Primers 1/4). DMSO-treated PfMSA180 (control)—980 bp, Rapamycin-treated—783 bp. Absence of bands in lanes 1 and 2 following rapamycin treatment confirms successful gene excision. Full length picture of the agarose gel showing PCR product bands and DNA ladder can be found in Supplementary Fig. 10.

Transgenic *P. falciparum* parasite lines were generated to allow conditional knockout of the *pfmsa180* gene using the rapamycin-mediated deletion in DiCre recombinase-carrying parasites^18^, to evaluate the function of PfMSA180. A one-step transfection approach with repair DNA homologous to the 5’ end of *pfmsa180* and incorporating two loxPint sequences^28^ in an out-of-frame arrangement was used. With a single guide RNA, a double strand break was created within the 5’ sequence of the open reading frame (ORF), and repair with the DNA containing the two loxPint sequences resulted in the successful generation of transgenic parasites, which were further cloned by limiting dilution (Supplementary Fig. 6). PCR with unique primer sequences was used to validate the modified parasites (Fig. 5a). Amplicon I with a size of 456 bp was amplified with primer pairs 1/2, amplicon II with a size of 535 bp was amplified with primers 3/4, and amplicon III was amplified with primers 1/4 with a size of 980 bp from DMSO-treated control parasites and 783 bp from rapamycin-treated parasites (Fig. 5b,c). The size of amplicon III from rapamycin-treated parasites (Fig. 5c) confirmed that recombination of the two loxP sites had occurred, resulting in the excision of 197 bp. This excision causes a reading frame shift and a premature stop codon, resulting in translation of a truncated 81 amino acid N-terminal sequence of MSA180 (Supplementary Fig. 7). This successful generation of a conditional *msa180* knockout parasite (denoted as i∆msa180) was further confirmed by immunoblotting with the polyclonal antibody preparations generated to the four recombinant proteins, against lysates of DMSO (WT) and rapamycin (iKO) treated parasites; PfMSA180 and its processed fragments were detected in the control parasites but not those treated with rapamycin (Fig. 2). Our design of a conditional MSA180 knockout *P. falciparum* parasite line differed from that used by Tan et al*.*^16^, although both approaches used the DiCre-recombinase technology. Whereas insertion of loxPint sequence between nucleotides 1252–1253 in Tan et al.^16^ resulted in a truncation of the *msa180* ORF with the potential of the first 417 amino acids still being expressed as a truncated protein, our approach resulted in a much shorter potential translation product of the N-terminal 81 amino acids. In both approaches the majority of the mature MSA180 protein is not being translated and the conserved C-terminus giving rise to a 48 kDa protein fragment after SUB1 cleavage is lacking.

**Figure 6:**
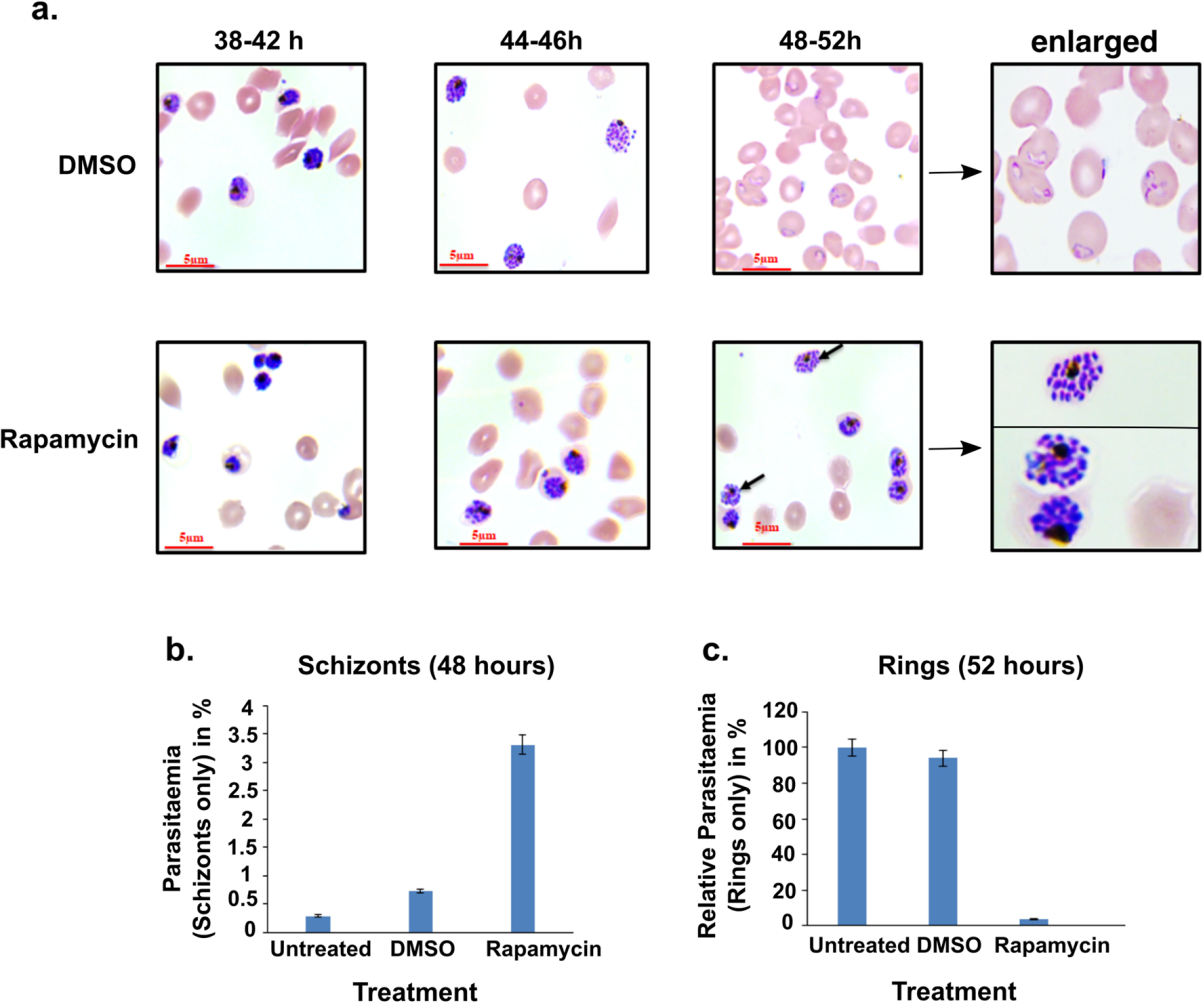
Induced deletion of *pfmsa180* results in the arrest of parasite egress at the end of schizogony. (**a**) Images of Giemsa-stained thin blood smears 38–52 h after rapamycin or DMSO treatment. The mock-treated (DMSO) parasites egress and invade new RBCs to form ring stages, while the rapamycin-treated parasites remain as late stage schizonts, often without a visible PVM and the merozoites filling the whole erythrocyte (arrows). The last panel displays a zoom-in image for rings and schizonts. Scale bar = 5 µm. (**b**) Number of schizonts observed in untreated, mock-treated or rapamycin-treated i∆msa180 parasites 48 h after setup (~ 3% parasitemia). The percentage schizonts observed in each treatment was calculated from three independent experiments. Error bars depict standard error of mean. (**c**) The relative percentage of rings observed in untreated, mock-treated or rapamycin-treated iΔmsa180 parasites 52 h after setup. The percentage of rings in each treatment was calculated from three independent experiments with respect to untreated parasites, for which the parasitemia was set to 100%. Error bars depict standard error of the mean of two independent experiments.

Having successfully generated *msa180* conditional knockout parasites we then tested their ability to grow in in vitro parasite cultures. Treating synchronised i∆msa180 cultures at ring stage with either DMSO or rapamycin, allowed development into schizonts as evidenced by Giemsa-stained thin blood films (Fig. 6a). However, further inspection of these Giemsa-stained blood films revealed that rapamycin-treated parasites displayed bulbous segmented schizonts at 48 h of asexual development, whereas in the DMSO-treated parasite schizonts had burst, releasing merozoites and allowing invasion and successful ring-stage formation (Fig. 6a). In the rapamycin-treated parasites, merozoites failed to egress, causing a dramatic loss of invasion and absence of ring-stage formation compared to the DMSO-treated parasites. Even after 52 h, the rapamycin-treated MSA180 knockout parasites failed to egress. Comparing the ratio of ring stages and schizonts at 48 h after treatment of i∆msa180 parasites with rapamycin or DMSO, it was clear that there were very few rings and a large number of schizonts in rapamycin-treated parasites, while in the control DMSO-treated parasite culture nearly all schizonts had burst and formed ring stage parasites (Fig. 6b,c), thus, suggesting a key role of MSA180 during merozoite egress. A close inspection of the Giemsa-stained smears suggested that in a significant proportion of the schizonts arrested by rapamycin treatment, the merozoites were dispersed throughout the host erythrocyte (Fig. 6a), indicating blockade of egress in the MSA180 knockout parasites.

## Discussion

*Plasmodium falciparum* is responsible for the severe and life-threatening complications of malaria. The genome contains about 5600 genes that are expressed during its life cycle, with many genes encoding hypothetical proteins with very little homology to those of other organisms^9^. The transcriptome and proteome profiles have provided insights for the identification of novel drug and vaccine candidates. For example, proteins that are accessible to plasma antibody represent putative vaccine candidates, particularly if specific antibodies interfere with or block essential steps such as merozoite invasion of erythrocytes^29^. Erythrocyte invasion is central to malaria pathogenesis, and mediated by a large invasion machinery involving multiple parasite proteins. Proteins of the merozoite surface, the pellicle and the apical complex, including the rhoptry, microneme and dense granules secretory organelles play a pivotal role in mediating the invasion process^30^. Based on transcriptomic expression profiling, *P. falciparum* genes have been divided into 15 clusters. 65% of the genes in cluster 15 are known to code for proteins involved in red cell invasion, implicating the remaining uncharacterised genes present in cluster 15 in the same process^12^. The increased expression of these genes typically begins ~ 38 h into the 48 h intraerythrocytic development of the asexual blood-stages. In this study, we have explored the role of PfMSA180, coded by the gene PF3D7_1014100, and selected on the criteria of transcriptome profiling and bioinformatic analysis for further functional characterisation as a potential target for the development of therapeutic intervention strategies. The *P. vivax* homologue, PvMSA180 was reported to be localized on the surface of merozoites, and found to be immunogenic during natural infection, eliciting humoral immune responses^14^. *P. vivax* MSA180 was proposed to be involved in merozoite attachment to the erythrocyte during red cell invasion^14^. Recently, whilst this study was underway, Nagaoka et al.^15^. showed that PfMSA180 is a merozoite surface antigen and a potential target of naturally acquired protective immunity to *P. falciparum* malaria. Additionally they showed that PfMSA180 is processed during schizogony, binds to the merozoite surface and interacts with the erythrocyte receptor CD47. Antibodies blocking the PfMSA180-CD47 interaction showed some potential to inhibit invasion^15^.

MSA180 is a conserved protein with homologs present in all *Plasmodium* species causing malaria in humans, as well as other species that infect rodents and other primates. The conservation of MSA180 across *Plasmodium* species suggests it plays a significant functional role in parasite biology. To elucidate this role, we expressed the protein (four constructs spanning the antigen) in recombinant form to raise specific antibodies to perform functional assays. The antibodies identified a high molecular mass protein (~ 180 kDa), likely to be PfMSA180, in *P. falciparum* 3D7 lysates. The antibodies also reacted with several smaller polypeptides, which may represent protein fragments, resulting from the proteolytic processing of the protein as suggested in the earlier study^15^ as well as a more recent study^16^. The absence of the high molecular mass band and smaller polypeptides following rapamycin treatment confirms that these proteins are PfMSA180 derived and that our antibodies are specific (Fig. 2). A number of the lower molecular mass bands may represent processing products resulting from cleavage at the three proposed SUB1 sites identified in MSA180 (such as bands of 125, 100, 96, 75, 63 and 48 kDa). However, apart from SUB1, other proteases may also be involved in proteolytic cleavage of PfMSA180, or artefactual bands might have resulted from lysis-induced proteolysis. By immunofluorescence microscopy, PfMSA180 appears to partially co-localise in the PV with MSP-1 at the merozoite surface in intact schizonts, although no transmembrane regions or GPI-anchor are predicted in MSA180. In a very recent study published whilst this manuscript was under review, Tan et al.^16^ showed that an epitope-tagged MSA180-HA was mostly expressed as a soluble protein, however, a small proportion of the protein was consistently observed in the membrane-associated fraction. Whether the mainly soluble nature is a consequence of the addition of the epitope tag to the relatively hydrophobic C-terminus of MSA180 is currently unknown. Using expansion microscopy on mature *P. falciparum* schizonts during different timepoints in the process of egress would be helpful to investigate the spatial distribution of SERA6, MSA180, various erythrocyte cytoskeleton components, PVM and the erythrocyte plasmalemma more clearly.

The antibodies raised in our study against the recombinant proteins prepared in *E. coli* reacted specifically with the native MSA180 parasite protein; however they did not inhibit merozoite invasion of erythrocytes (< 5% inhibition of growth at 10 mg/ml IgG in in vitro culture). Our results therefore suggest that PfMSA180 is not a vaccine candidate, a conclusion consistent with the recently published data from Nagoaka et al*.*^15^, which also failed to support the idea that PfMSA180-specific antibodies potently inhibit parasite invasion (21.3% inhibition at 20 mg/ml). We also examined whether the recombinant proteins bound to the surface of erythrocytes using a FACS-based assay. No binding activity was detected, which is contrary to the earlier report of erythrocyte binding by PfMSA180 from parasite culture supernatant. It is possible that the full-length native protein folds into a three dimensional conformation that confers erythrocyte binding activity, or that the recombinantly produced protein fragments fold differently to the native protein. In conclusion, from this part of the study, we have shown that PfMSA180 appears to be associated with the merozoite surface in the PV space of developing schizonts and is likely subjected to processing during the asexual blood-stages of the life cycle, but it does not appear to be a good vaccine candidate, given that antibodies did not, or only very poorly inhibited invasion.

A previous piggyBac transposon mutagenesis study identified *pfmsa180* gene to have an essential role in the parasite^8^, but this approach provided no information on when its function is important. Therefore, to elucidate the functional role of PfMSA180, parasite lines with an inducible *pfmsa180* gene knockout were generated using the CRISPR/Cas9-based technology to introduce a rapamycin-inducible DiCre recombinase dependent deletion^18,31^. As *pfmsa180* lacks introns, we inserted loxP sites within *sera2* introns (loxPint) as previously described^28^. By insertion of two loxPint sequences separated by 94 bp of recodonised sequence, we generated a transgenic parasite containing the *msa180* gene with loxP sites. Addition of rapamycin produced functional Cre recombinase and excision of the floxed piece of DNA between the two loxP sequences, leading to a premature termination of translation and a truncated protein. Following rapamycin treatment at early ring stages, parasite development was arrested at a late schizont stage. Merozoites that had formed failed to egress from the erythrocyte even 4 h after normal egress at 48 h (a total of 52 h) after rapamycin treatment, clearly implicating MSA180 in this crucial step of merozoite release prior to invasion of a new red blood cell, although formally an additional role in invasion cannot be excluded. Egress of merozoites from the multinucleate schizont is mediated in part by several serine and cysteine proteases, which are responsible for the destabilisation and disruption of both the PVM within which the merozoites reside^32^, as well as the erythrocyte plasma membrane. *P. falciparum* SUB1 is an essential protease released into the PV from exonemes, which mediates the proteolytic maturation of several papain-like proteases from the SERA family to trigger the process of merozoite egress^23^. PfSUB1 is also responsible for the proteolytic maturation of merozoite surface proteins MSP-1, MSP-6 and MSP-7^21^. By analogy, PfMSA180 also undergoes proteolytic processing by PfSUB1 as it possesses several predicted PfSUB1 sites, but this suggestion still needs to be further confirmed experimentally. Recently Tan et al.^16^ used a genetic ablation and epitope-tagging strategy to show that MSA180 is involved in parasite egress. They also showed that two protein fragments of MSA180 mapping to the central and C-terminal region of MSA180 co-immunoprecipitated with SERA6, a protease that is essential for cleavage of components of the erythrocyte cytoskeleton and parasite egress. The genetic disruption of MSA180 blocked parasite egress, demonstrating a phenotype identical to the one seen with SERA6-inducible knockout parasites previously. The authors observed that in ∆MSA180 parasites, the maturation of SERA6 protease is hindered and concluded that MSA180 plays a crucial part in the autoproteolytic processing of SERA6^16^, ultimately facilitating merozoite egress. We failed to identify SERA6 as an interaction partner for MSA180 in our immunoprecipitations, perhaps because this interaction seems to take place only in the final stages of egress, and it is likely that the parasites used here without treatment using egress inhibitors were not mature enough. Also the exact processing sites in PfMSA180 in addition to the SUB1 site at residue 1079 and their relevance, as well as MSA180’s potential interactions with other proteins besides SERA6, such as those that might mediate binding to the merozoite surface or host cell cytoskeleton, warrant further investigations.

In conclusion, our study indicates that PfMSA180 has an essential role in merozoite egress from the erythrocyte during the asexual blood stages of the parasite life cycle, a conclusion shared by Tan et al.^16^. In contrast, we found no evidence that it was important in erythrocyte invasion, either from the use of antibodies or from direct binding studies, however we cannot formally exclude additional functions during invasion, similar to what has been reported for MSP-1^33^. Our evidence, however, does not support any potential for PfMSA180 as a vaccine candidate. The molecular basis of the role of PfMSA180 in egress, especially its proposed interaction and activation of SERA6, remains to be further established but it is possible that this process may be inhibitable by suitable small molecules as demonstrated in Tan et al*.*^16^ and is therefore a potential target for the development of new medicines against malaria.

## Methods

### Preparation of PfMSA180 recombinant proteins

Construct 1: The DNA for the N-terminal recombinant protein construct 1 (C1) was cloned in the pET24b vector using NdeI and XhoI sites. The selected plasmid was inserted into *E. coli* ArcticExpress cells and expression of a soluble protein was induced following treatment with 1 mM isopropyl-1-thio-β-D-galactopyranoside (IPTG). Following induction, bacteria were harvested by centrifugation and the cell pellet was lysed by sonication. The protein was purified from the supernatant fraction of the lysate by immobilized metal affinity chromatography (IMAC) using a Ni–NTA column and eluted with increasing imidazole concentration. The protein was purified by size exclusion chromatography using a S200 16/600 column (GE Healthcare).

Constructs 2, 3 and 4: The DNA for construct 2 (C2) and 3 (C3) was cloned in the pET24b vector using NdeI and XhoI sites, while the C-terminal construct 4 (C4) was cloned in pET24b vector using NheI and XhoI sites. *E. coli* Shuffle 26 cells were transformed with the C2 and C3 plasmids while the *E. coli* BLR cells were transformed with the C4 plasmid. Following maximal expression induced by treatment with 1 mM IPTG, the recombinant proteins (C2, C3, C4) were expressed in the form of inclusion bodies (IB). The bacterial pellet was lysed by sonication and the inclusion bodies were prepared and washed thoroughly with buffers containing Triton X-100 and Urea. Washed IBs were solubilised in a buffer containing 6 M guanidine hydrochloride and the proteins were purified by IMAC using Ni–NTA columns and elution done with increasing imidazole concentration. The IMAC purified proteins were refolded in a Tris-based buffer (55 mM Tris pH 8.2, 264 mM NaCl, 11 mM KCl, 2.2 mM MgCl_2_, 2.2 mM CaCl_2_, 550 mM L-Arginine, 440 mM sucrose) and dialysed against a Tris-based buffer (55 mM Tris pH 8.2, 10.56 mM NaCl, 0.44 mM KCl, 5% Glycerol). All three proteins were purified to homogeneity by size exclusion chromatography using a S200 16/600 column (GE Healthcare).

### Animal immunization and antibody purification

BALB/c mice were injected with recombinant antigen to raise specific antibodies. For priming (day 0), 25 µg of antigen formulated with complete Freund’s adjuvant (CFA) (Sigma, St. Louis, MO) was injected intramuscularly, then two booster immunizations with 25 µg of antigen formulated with incomplete Freund’s adjuvant (IFA) (Sigma, St. Louis, MO) were injected on days 28 and 56. Serum was collected on day zero before priming (pre-bleed), and on days 14, 42 and 70.

New Zealand White (NZW) rabbits were immunized intramuscularly with all four recombinant antigens. For priming (day 0), 100 µg of antigen formulated with CFA was injected, then booster immunizations with 100 µg of antigen formulated with IFA were given on days 28 and 56. Serum was collected on day zero before priming (pre-bleed), and at days 14, 42 and 70^34^.

Serum immunoglobulin G (IgG) was purified from the rabbit sera collected on Day 70 using a protein G-sepharose column (GE Healthcare). Serum was mixed with binding buffer (20 mM phosphate buffer, pH 7.0) in a 1:1 ratio and loaded on to a pre-equilibrated IgG column (1 ml packed column volume). After the diluted serum was loaded, the column was washed with binding buffer (30 times the column volume). IgG was eluted with elution buffer (0.1 M glycine–HCl, pH 2.7) and neutralised by addition of 5% neutralisation buffer (1 M Tris–HCl, pH 9.0). The purified IgG was pooled, concentrated (10 kDa Centricon, Merck) and dialysed against incomplete RPMI 1640 medium. The purified IgG was filtered with 0.2 µm syringe filters and stored at -80 °C until used for invasion inhibition assays^34^.

### Erythrocyte binding assay

10 µg of recombinant proteins were incubated with 100 µl of packed erythrocytes in incomplete RPMI 1640 (containing 360 μM hypoxanthine, 24 mM sodium bicarbonate and 10 μg/ml gentamycin) at 37 °C for 1 h. The erythrocytes were washed and incubated with primary antibody against the recombinant antigens at 37 °C for 1 h. The cells were washed and incubated with Alexafluor-594 (Sigma) conjugated secondary antibodies. The cells were washed again and protein binding was measured by flow cytometry (BD FACS Aria)^35^.

### Parasite culture

The *P. falciparum* 3D7 and II-3 DiCre-expressing parasite lines were cultured in vitro in complete RPMI 1640 medium (Invitrogen) containing 0.5% Albumax, 360 μM hypoxanthine, 24 mM sodium bicarbonate and 10 μg/ml gentamycin, in human O positive erythrocytes at 2% hematocrit in mixed gas (5% CO_2_, 5% O_2_, 90% N_2_) at 37 °C as described previously^36^. The parasites were synchronised by sorbitol^37^ and percoll treatments^38^.

### Growth inhibition assay (GIA)

The assay was performed as per the protocol described earlier^34,39^. Synchronized late-stage schizont parasites at 2% hematocrit and 0.3% initial parasitemia were incubated with purified IgG at 10.0 mg/ml (CyRPA was tested at 2.5 mg/ml). After one-cycle (40–44 h) of invasion, parasites were stained with ethidium-bromide and parasitemia was measured by flow cytometry (BD FACS Aria). Inhibition of parasite growth in the presence of IgG was calculated relative to the growth of parasites incubated with the IgG purified from pre-immune sera.

### Immunoblot analysis of parasite lysate

*P. falciparum* synchronised late-stage schizonts were harvested by centrifugation. Erythrocytes were lysed with 0.05% (w/v) saponin before the parasite pellet was lysed in RIPA buffer (Sigma) with 2X protease inhibitor cocktail (Roche) on ice for 2–3 h. The parasite lysate was clarified by centrifugation (16,000 g, 30 min) and proteins in the supernatant were resolved by 8% SDS-PAGE and transferred on to 0.45 µm nitrocellulose membrane overnight on ice at 20 V. The membrane was blocked in 5% skimmed milk and probed with MSA180 primary antibodies (1:250) and HRP-conjugated secondary antibody (1:5000). The blots were developed using ECL reagent (Thermo Fisher).

### Immunofluorescence assay

The slides for the late-stage schizonts were prepared by smearing infected erythrocytes onto glass slides. The slides were then air-dried and fixed by dipping in pre-chilled methanol for 30 min. The fixed slides were blocked with 3% BSA (Sigma Aldrich) prepared in PBS overnight at 4 °C, and then incubated with antibodies against the PfMSA180 recombinant proteins (1:100) along with marker antibodies (MSP-1, Exp-1, 1:200 dilution prepared in PBS) for 2 h at room temperature. The slides were washed vigorously with PBS containing 0.05% Tween 20 (PBST) and PBS and incubated with the corresponding secondary antibodies labelled with Alexa Fluor 488 and 594 (Thermo Fisher Scientific) for 1 h. The slides were then washed again with PBST and PBS, mounted in ProLong Gold antifade reagent and DAPI (Invitrogen) and viewed by confocal microscopy (Olympus FluoView™ FV1000) and processed using Imaris and NIS element.

### Immunoprecipitation

Synchronized *P. falciparum* 3D7 schizont stage parasites (44–48 h), were harvested by centrifugation at 2,000* g* for 5 min. The erythrocytes were lysed by incubating the pellet in 0.15% saponin for 10 min on ice. Immunoprecipitation was performed using the Pierce Co-Immunoprecipitation kit. The parasite material was lysed on ice by adding an equal volume of cell lysis buffer and 2X Protease inhibitor cocktail (Roche) for 3 h. The lysate was collected after centrifugation at 15,000* g* for 30 min at 4 °C^40^. The lysate was cleared by incubation with control agarose resin for 1 h at room temperature with a gentle end-to-end mixing. The pre-cleared lysate was collected following centrifugation at 1,000* g* for 1 min. In the meantime, IgG from day 70 sera was bound and cross-linked to protein A/G agarose resin. 2 mg of the pre-cleared schizont lysate was incubated with antibodies crosslinked to the resins overnight at 4 °C. The column was washed and the immunoprecipitated samples were eluted with elution buffer. The samples were trypsin digested and analysed by mass spectrometry (V proteomics, New Delhi, India).

### Generation of inducible gene knockout parasites

Identification of guide RNA (gRNA) sequence and insertion into CRISPR/Cas9 plasmids: The gRNA sequences were identified using bioinformatic tools (protospacer and CRISPR guide RNA/DNA design tool at EuPaGDT^41^). The identified gRNA sequences were procured from Sigma. The gRNA oligonucleotides were phosphorylated at 37 °C for 30 min using T4 polynucleotide kinase (NEB) and annealed by a temperature ramp down from 94 °C to 25 °C at 5 °C/min. The Cas9 vector (pDC2-Cas9-hDHFRyFCU)^18^ was digested at 37 °C with BbsI restriction enzyme. The digested vector was dephosphorylated with calf intestinal alkaline phosphatase (Roche) at 37 °C for 30 min followed by incubation at 50 °C for 30 min. The phosphatase was deactivated by adding EDTA and incubating at 75 °C. The digested vector was column purified (QIAquick PCR purification kit, Qiagen).

The annealed oligonucleotides were stored at 4 °C and then diluted with nuclease free water in a 1:200 ratio and ligated with the digested vector. The ligation mixture was transformed into XL10 gold competent cells and insertion of the gRNA into plasmids was confirmed by sequencing of miniprep plasmid DNA (Qiagen Miniprep kit). Midi plasmid preparation was performed for selected clones (Qiagen midi kit).

Design of repair plasmids (used by the parasites for homologous DNA repair after Cas9 double strand DNA cleavage): Since the *pfmsa180* gene lacks introns, loxP sequences were inserted using the loxPint approach^28^. DNA sequence between the two loxP sites was recodonised and synthesized by GeneArt. The repair plasmids were transformed into XL10 gold competent cells and maxi plasmid preparations were prepared (Qiagen Maxi Kit). The repair plasmids were designed with a suitable restriction enzyme site at either end.

To create the plasmid mixture for transfection of *P. falciparum* II-3 schizonts, the plasmid was linearized by restriction enzyme digestion and mixed with Cas9-guide RNA plasmid at a molar ratio of 5:1, and then ethanol precipitated at − 20 °C overnight. The DNA precipitate was collected by centrifugation at 13,000* g* for 30 min at 4 °C; the ethanol was decanted and the pellet air dried. The DNA was dissolved in 10 µl Tris–EDTA buffer and stored at − 20 °C.

Parasite culture and transfection: Schizont stages of the II-3 parasite were obtained from tightly synchronised cultures by percoll purification, and electroporated with the DNA mix (Amaxa 4D-Nucleofector using P3 Primary Cell Kit). Following transfection, the cultures were treated with 2.5 nM WR99210 for 4 days of selection and then cultured further without drug until viable parasites reappeared (10–14 days). Genomic DNA was isolated (Qiagen genomic DNA isolation kit) and analysed by PCR for integration. Marker-free parasite clones were generated seven weeks post transfection by limiting dilution cloning. These were tested by adding 10 nM rapamycin to early ring stage parasites to allow DiCre recombinase-mediated excision of DNA^18^. DNA was isolated from the rapamycin treated and control (DMSO-treated) samples (Qiagen) and excision was monitored by PCR.

### Statistical analysis

The results were analysed using MS Excel and reported as mean ± Standard error post data preparation.

### Ethics declarations

The animal experiments were approved by Jawaharlal Nehru University (JNU) Institutional Animal Ethics Committee (IAEC Code No: 09/2019) as described by the ARRIVE guidelines (PLoS Bio 8(6), e1000412,2010). The use of transgenic parasites was approved by JNU Institutional Biosafety Committee (IBSC No: JNU/IBSC/2020/52) as per the guidelines of Department of Biotechnology, Government of India. The transgenic parasites were generated at The Francis Crick Institute. All methods were performed in accordance with the relevant guidelines and regulations.

## Supplementary Information

Supplementary Information.

## Data Availability

All relevant data files generated for this study have been added to this manuscript (main text or the supplementary file). Reagents generated as part of this study are available from the authors upon reasonable request.
