# Supplementary material for: Genetic disruption of Plasmodium falciparum Merozoite surface antigen 180 (PfMSA180) suggests an essential role during parasite egress from erythrocytes
Source: Sci Rep. 2021 Sep 28;11:19183. doi: 10.1038/s41598-021-98707-0 (PMC8479079; doi:10.1038/s41598-021-98707-0)
Supplement: Supplementary file 1 — Supplementary Information. [file 41598_2021_98707_MOESM1_ESM.pdf]

Supplementary Information

Genetic disruption of *Plasmodium falciparum* Merozoite Surface Antigen 180 (PfMSA180) suggests an essential role during parasite egress from erythrocytes

Vanndita Bahl[1¶]*, Kritika Chaddha[1¶], Syed Yusuf Mian[1], Anthony A. Holder[2], Ellen Knuepfer[2,3]*, Deepak Gaur[1]

[1] Laboratory of Malaria and Vaccine Research, School of Biotechnology, Jawaharlal Nehru University, New Delhi, India

[2] Malaria Parasitology Laboratory, The Francis Crick Institute, 1 Midland Road, London, NW1 1AT, United Kingdom

[3] The Royal Veterinary College, Hawkshead Lane, North Mymms, Hatfield, Hertfordshire AL9 7TA, United Kingdom

¶ These authors contributed equally

*Corresponding authors

Please send correspondence to:

Dr Ellen Knuepfer
The Royal Veterinary College
Hawkshead Lane
Hatfield, AL9 7TA, United Kingdom
Email: eknuepfer@rvc.ac.uk

Miss Vanndita Bahl
School of Biotechnology
Jawaharlal Nehru University
New Delhi India 110067
E-mail: vandy.bahl@gmail.com

Figure Legends

Supplementary Figure 1: PfMSA180 is a conserved protein in *Plasmodium* species causing human malaria. PF3D7- *P. falciparum* 3D7, PmUG01- *P. malariae* UG01, PocGH01- *P. ovale curtisi* GH01, PKNH- *P. knowlesi* strain H, PVP01- *P. vivax* P01. Multiple sequence alignment revealed most conserved residues are in the N-terminal region: residues 1-163, central region: residues 417-731 and C-terminal region: residues 1085-1455. The C-terminal region contains most identical residues (~37%). "*" identical residues; ":" conserved substitutions; "." semi-conserved substitutions.

Supplementary Figure 2: Immobilised Metal Affinity Chromatography (IMAC) purification of PfMSA180 recombinant proteins. PL- Total lysate, FT- flow through from IMAC column, W1- 10mM imidazole, W2- 20mM imidazole; elution profiles using increasing concentrations of imidazole (50 mM - 500 mM), collected each in 2ml fractions.
(a) Construct 1 (C1) was expressed as soluble protein and was purified from a bacterial pellet supernatant obtained after sonication; **(b)** Construct 2 (C2), **(c)** Construct 3 (C3) and **(d)** Construct 4 (C4) were expressed in inclusion bodies and the protein was purified from these inclusion bodies obtained after sonication.

Supplementary Figure 3: Measurement of the antibody responses in rabbits against recombinant PfMSA180 proteins. (a) C1 PfMSA180, **(b)** C2 PfMSA180, **(c)** C3 PfMSA180, **(d)** C4 PfMSA180. Immunogenicity of recombinant PfMSA180 proteins was analyzed by ELISA. The serum samples were serially diluted and end point titers were assessed. Pre-immune sera were used as controls. High titer antibodies were obtained against all recombinant PfMSA180 proteins. The error bars represent the standard error of the mean.

Supplementary Figure 4: Measurement of the antibody responses in mice against recombinant PfMSA180 proteins. (a) C1 PfMSA180, **(b)** C2 PfMSA180, **(c)** C3 PfMSA180, **(d)** C4 PfMSA180. Immunogenicity of recombinant PfMSA180 proteins was analyzed by ELISA. The serum samples were serially diluted and end point titers were assessed. Pre-immune sera were used as controls. High titer antibodies were obtained against all recombinant PfMSA180 proteins. The error bars represent the standard error of the mean.

Supplementary Figure 5: Putative PfSUB1 processing sites in PfMSA180. The high molecular weight parasite protein MSA180 is predicted to possess potential PfSUB-1 proteolytic cleavage sites[1]. The three potential PfSUB1 cleavage sites are depicted along with the predicted MSA180 processing fragments.

Supplementary Figure 6: Schematic representation of the generation of inducible *pfmsa180* gene knockout parasites.

(a) DiCre recombinase-expressing *P. falciparum* parasites II-3 were transfected with pDC2-Cas9-hDHFRyFCU[2] with gRNA (guide RNA) sequence and a repair DNA plasmid carrying two loxPint sequences (SERA2 intron with a 34 bp lox P site)[3] flanking a 94 bp recodonised ORF segment and having 5' homology region of 271 bp and a 3' homology region of 303 bp. The selected gRNA sequence targets Cas9 to a sequence close to the 5' end of the ORF.

(b) Homolgous DNA repair utilises the linearised repair plasmid as homology template resulting in the insertion of the two loxPint sequences flanking the 94 bp recodonised ORF sequence. On addition of rapamycin a functional DiCre-recombinase catalyses the recombination between the two loxP sites resulting in the deletion of 197 bp including the recodonised ORF fragment. This excision changes the reading frame resulting in early termination of translation for PfMSA180.

Supplementary Figure 7: Transgenic *pfmsa180* DNA sequence.

(a) Part of the DNA sequence of the transgenic *msa180* ORF before excision: The *pfmsa180* ORF with the loxPint (green) and recodonised piece of DNA (Yellow) flanked by two loxPint sites.

(b) Part of the DNA sequence of the transgenic *msa180* ORF after excision: Rapamycin induced excision leads to the removal of one loxPint and the piece of recodonised DNA.

(c) Generation of premature STOP codon after excision: After excision the frame shift in DNA sequence results in the truncation of MSA180 at amino acid 81. Codons are depicted here including the premature stop codon TAG (Red).

Supplementary Figure 8: Purified PfMSA180 recombinant protein fragments (C1-4).

(a) Purified recombinant PfMSA180 products (C1-4) separated by SDS-PAGE were stained with coomassie brilliant blue. Ni-NTA elutes were pooled, dialysed and concentrated. M-Protein marker, BC- Before concentration, AC- After concentration. 1-7 correspond to

collected fractions of elutes of the purified antigen. The dashed rectangles represent the area of cropped gel images shown in Figure 1b.

(b) Immunoblots stained with anti- hexa-histidine tag antibody followed by secondary alkaline phosphatase-conjugated secondary antibody. The dashed rectangles represent the area of cropped immunoblot images shown in Figure 1b.

Supplementary Figure 9: Expression of PfMSA180 in wild-type but not conditional MSA180 knockout parasites.

Full size immunoblots showing a high molecular weight antigen and its processed protein fragments in wild type parasites (WT), whilst bands are absent in the inducible knockout (iKO) parasites (after rapamycin treatment). Immunoblots were probed with MSA180-specific antibodies raised against recombinant MSA180 (a) Construct 1, (b) Construct 2, (c) Construct 3 and (d) Construct 4. (e) Immunoblot probed with anti-CyRPA antibodies were used as a positive loading control. The dashed rectangles represent areas of the immunoblots shown in Figure 2.

Supplementary Figure 10: PCR to confirm integration of the repair plasmid into the *pfmsa180* locus.

(a) Full size agarose gel picture showing diagnostic PCR products. Presence of correctly sized bands in lane I and II confirms integration of repair the plasmid (including a floxed 94 bp recodonised DNA sequence) in one transgenic parasite clone (clone 1) and differentiates it from wild type parasites (II-3 DiCre). Product sizes in lanes III between clone 1 and wild type parasites (II-3 DiCre) indicate the larger size of 980 bp after integration of the loxP-flanked repair DNA fragments, compared to 774 bp in the wild type parasites. The dashed rectangle represents the part of the agarose gel image shown in Figure 5b.

(b) Full size agarose gel picture showing PCR analysis of successful excision of a floxed recodonised *pfmsa180* sequence after the addition of rapamycin. C- Control DMSO treated parasites, R- Rapamycin treated parasites. Reduction of PCR band size in Lane III of ‘R’ compared to ‘C’ confirms the successful deletion of the floxed piece of DNA after rapamycin treatment. For band sizes please see Figure 5. The dashed rectangle represents part of the agarose gel image shown in Figure 5c.

Supplementary Table 1: List of primers used for the cloning of PfMSA180 constructs and for the conditional gene knockout study

**Primer**	**Sequence**
MSA180C1FP	CTATAGGGCCTTCGTGCATATGAATGAGAAAAATAGGAAAGCTATT
MSA180C1RP	CTATAGGGTTATAGTCTCGAGAGTTGAATTGGGTGACGAA
MSA180C2FP	GTATACCTGAACTTGCATATGAACAAAGAATCTTTAATCTTTCC
MSA180C2RP	GCTTGTGGCTCGAGATTATATATATTTTTTGGATCATC
MSA180C3FP	CATGTATACGAATGCCATATGAATAATGTACACGATACAGC
MSA180C3RP	ACGCTTGGCTCGAGATCATTTTGTTCAGTGTTAG
MSA180C4FP	GTATGTAATGTGGCTAGCAATAAGGAAGAGGATATGAATG
MSA180C4RP	CTGACTACGTGCTCGAGATTTCTAAAATCTAGTGCATC
MSA180NextFP	ATGGTTCATTGTCCCTTTTTGTAGTG
MSA180NextRP	GGCAAAGGGTTTGTTTGATAAGGG
MSA180NintFP	GAAGTTCGATAAGTTTATAGACGAGTTC
MSA180NintRP	TATCGAACTTCTTAGAGTCTTTAGGG
MSA180_185guideF	ATTGGAAGAATTAAAGTATGCAT
MSA180_185guideR	AAACATGCATACTTTAATTCTTC

Supplementary Table 2: Immunoprecipitation by PfMSA180 polyclonal antibodies.

The antibodies were raised to PfMSA180 Construct 3 (C3) and Construct 4 (C4) and a significant number of peptides derived from MSA180 were observed. The Mass Spectrometry analysis was searched against the Plasmodium database on Uniprot[4].

Table 2a: List of proteins identified by the polyclonal antibodies

Accession	Description	Unique peptides	PSM
Q8IJQ4	Uncharacterized protein PF3D7_1014100	53	190
Q8I0U8	Merozoite surface protein 1 PF3D7_0930300	45	136
C6KTB4	Acetyl-CoA synthetase, putative PF3D7_0627800	32	71
Q8I0V3	60 kDa chaperonin PF3D7_1232100	21	63
Q8IE67	Phosphoribosylpyrophosphate synthetase PF3D7_1325100	15	41
Q8IKF0	Eukaryotic initiation factor 4A PF3D7_1468700	10	35
Q8IKH8	40S ribosomal protein S3 PF3D7_1465900	8	27
Q8IAX5	40S ribosomal protein S16, putative PF3D7_0813900	6	24
O97266	Eukaryotic translation initiation factor 4E PF3D7_0315100	7	22
C6KT18	Histone H2A PF3D7_0617800	3	17
Q8I542	Calcyclin binding protein, PF3D7_1238100	5	10
Q8IIX0	60S acidic ribosomal protein P1, putative PF3D7_1103100	2	7

Table 2b: List of PfMSA180 Peptides detected in immunoprecipitates with polyclonal antibodies

**Peptides Detected with C3 Antibody**
IKGNSEEFSFDNELPEQTESFPLNKPQDHEAFYNLK
IHNILKDFNINENIMTNK
IKGNSEEFSFDNELPEQTESFPLNKPQDHEAFYNLKK
SAIDKYVHYEYKR
GNSEEFSFDNELPEQTESFPLNKPQDHEAFYNLK
KIANTIYVNVGQSGINGFFNFFDFREK
KHHTNVYEPNDEEKQNEQK
NIYNMNNVHDTAYYHNSR
LTNNFKENDEGLKNENNINNNEDNQNDNMNIVLGK
NLTEFLENTER
NFYNISNENGDNTFNNNNNNMDNK
NFYNISNENGDNTFNNNNNNMDNKKR
MNGKLPIDDPKNIYNMNNVHDTAYYHNSR
NHMMLSNEQFINKNK
NNSETNENISESNSGNPELNNENSYSVK
LSYFNLPSLK
INYIFFNYIPLENYVNNGDALDFR
VTGDSVENINEQTNNNQYPNTEYNTIQR
VGDQFFPTYSNLGK
VGDQFFPTYSNLGKDDHDLEHSAK
ELAEISTSNLFYPKKDIILR
ELAEISTSNLFYPKK
ELAEISTSNLFYPK
DNNYYYNNSDNNNYNER
DMPSLEDNFYEHLKYPDINTIHIYYNASPVK
ALLQQSNKDTPIHK
YFPTKDMPSLEDNFYEHLKYPDINTIHIYYNASPVK
YKDNNYYYNNSDNNNYNER
NLTEFLENTERINTFVR
NHMMLSNEQFINK
YPDINTIHIYYNASPVK
YMAENKFNLPMSSEVENK
NYKNLTEFLENTER
VDVIDEK
TIIIDEIKSK
ENDEGLKNENNINNNEDNQNDNMNIVLGK
ISENLNR
LPIDDPKNIYNMNNVHDTAYYHNSR
DMPSLEDNFYEHLKYPDINTIHIYYNASPVKLNEVNDLK
DMQGNNIKIEQNK
ANQQFFSYK
MNEFDYINNFSASYLLNQLIIFQDKFNYIK

ITSDILYK
FIPINAFITLENK
IFYINSYR
MVNDTWITPYAFVVYSK
HHTNVYEPNDEEKQNEQK
NHMMLSNEQFINKNKYAK
FNLPMSSEVENK
SAIDKYVHYEYK
**Peptides Detected with C4 Antibody**
IKGNSEEFSFDNELPEQTESFPLNKPQDHEAFYNLK
IHNILKDFNINENIMTNK
SAIDKYVHYEYKR
GNSEEFSFDNELPEQTESFPLNKPQDHEAFYNLK
NIYNMNNVHDTAYYHNSR
NLTEFLENTER
NFYNISNENGDNTFNNNNNNMDNK
NFYNISNENGDNTFNNNNNNMDNKKR
LNEVNDLK
INYIFFNYIPLENYVNNGDALDFR
VTGDSVENINEQTNNNQYPNTEYNTIQR
VGDQFFPTYSNLGK
VGDQFFPTYSNLGKDDHDLEHSAK
ELAEISTSNLFYPKKDIILR
ELAEISTSNLFYPKK
ELAEISTSNLFYPK
DMPSLEDNFYEHLKYPDINTIHIYYNASPVK
YKDNNYYYNNSDNNNYNER
NLTEFLENTERINTFVR
NHMMLSNEQFINK
NYKNLTEFLENTER
VDVIDEK
TIIIDEIKSK
ENDEGLKNENNINNNEDNQNDNMNIVLGK
ISENLNR
DFNINENIMTNK
ANQQFFSYK
MNGKLPIDDPK
ITSDILYK
FIPINAFITLENK
IFYINSYR
FNLPMSSEVENK

References:

1. Withers-martinez, C. *et al.* Plasmodium subtilisin-like protease 1 (SUB1): Insights into the active-site structure , specificity and function of a pan-malaria drug target. *Int. J. Parasitol.* **42**, 597–612 (2012).
2. Knuepfer, E., Napiorkowska, M., Ooij, C. Van & Holder, A. A. Generating conditional gene knockouts in Plasmodium – a toolkit to produce stable DiCre recombinase-expressing parasite lines using CRISPR / Cas9. Sci. Rep. 1–12 (2017). doi:10.1038/s41598-017-03984-3
3. Jones, M. L. *et al.* A versatile strategy for rapid conditional genome engineering using loxP sites in a small synthetic intron in Plasmodium falciparum. *Sci. Rep.* 1–9 (2016). doi:10.1038/srep21800
4. Consortium, T. U. The Universal Protein Resource (UniProt). **36**, 190–195 (2008).

Supplementary Figure 1:

```
PF3D7   MNRIFYFCLFTILFWLSLVSGENVNNKNCNEKNRKAILLALLKNSLVDNKDYNNSEELKY 60
PmUG01  MLRIAYFSLSFIILSFLFFSGHNAL-PNEEDKNKKAILLALLTNTFINNKEYKNGEDINI 59
PocGH01 MLRIIYFSFFPILFSLFLISGHDAI-SNVEDKTKKAILLALLKNTFIDNEEYKEPNDLNN 59
PKNH    MSRITFLFSLSILFFFFLLPGQNAL-TIDDDKNKRATLLALLKNTFIDNKGNKKSDDIKG 59
PVP01   MPRITPLFLLSILLSFFLFSGQNAL-TNDDDTNKRATLLALLKNTFIDNTENKKPDDINT 59
        * **  :    *:: : :. *.:.     ::..::* *****.*::::*   :: ::::

PF3D7   ALEHIQNSELYPKDSKKFDKFIDEFFSYYNIHVNFTDEEKRILHISGVFKEFYVDVDNLN 120
PmUG01  ALENINNMKLHPTDNDKFDKFLDALFKHHNIYVTLDDHDKRIIHISGVLSEFYVDVDTLT 119
PocGH01 ALENINNMNIHPTDNKKFDNFLEELFKHYNVHVTFSDMDKRILHTSGVFNDIYVDVNSLD 119
PKNH    ALENIKNMTLQPTDTDKFDKFLDQFLKFFQIYVTFSDKDKRVLHLSGVLNDVYVDVDSLS 119
PVP01   ALENINNMTLHPTDTDKFNKFLDHFLKFFHIYVSFSDKDKRVLHLSGVLNEVYVDVESLS 119
        ***:*:*  : *.*..**::*:: ::...:::*.: * :**::* ***:.:.****:.*

PF3D7   KDEMKEYFKKNYEKGLSLINLIVHSNLIIQQFDHDIIDKKKVH----------------- 163
PmUG01  EEKQKEYFNTFYEKGHTLINLILHSNLIHTKHDKDVNKENHKEETKPNHPDSPIPDEEPN 179
PocGH01 KGNTKEYFNGIHKKALSLINLVLHSNLVHPKYAEINIEKGSQTGNDLIQENS------QD 173
PKNH    KENLQKHFDSLYEKGLNLINLIVHSNLVHPKYDETEMHGVDAEEKDH------------- 166
PVP01   EENLQKHFDSVHEKGLNLINLIVHSNLVHPKYDETAVQGMEGEEPEQ------------- 166
        : : :::*.  ::*. .****::****:  :. .   .

PF3D7   ------------------------------------------------------------ 163
PmUG01  LEKEPIPDDVSKGEVNHQLNYNNVYDSDIEVESNHEVEPNHEVEPNHAVEPNHAVEPNHA 239
PocGH01 TEG------EPKGN------------------TGEGHEPVYDFKNNQGGEPDNAYGHI-- 207
PKNH    VS---------------------------------------------------------- 168
PVP01   TDG------PTEGP------------------GSHG-----KVDPPQEGDPQEEEGPSQE 197


PF3D7   ------------------------------------------------------------ 163
PmUG01  VEPNHAVEPNHATEPNHATEPNPENEPIPDDVSKGEVNHQLNYNNVYDSDIEVESNHEVE 299
PocGH01 ------------------------------------------------------------ 207
PKNH    ------------------------------------------------------------ 168
PVP01   GHPQEK------------------------------------------------------ 203


PF3D7   ------------------------------------------------------------ 163
PmUG01  PNHEVEPNHAVEPNHAVEPNHATEPNHATEPNHATEPNHAVEPNHATEPNPENEPNPENE 359
PocGH01 --------QGEEPVHEYEYNQGGEPDNACEHIQGEEPVH---EY---EY----------N 243
PKNH    ---------------------------DNYAENIKGPAHYEESQ---EY----------E 188
PVP01   ----VDPTQENHPHEKVDPPQEGDPAQESHPHQKDGPAQQDHSQ---EY----------A 246


PF3D7   ------------------------------------------------------------ 163
PmUG01  PIPDDVSKGEVNHQLNYNNVYDSDIEVEPNHAAEPNPENEPIPDDVSKGE-VNHQLNYNN 418
PocGH01 QGE------EPVHEYEYNQG------EEP-----------VHEYEYSQGEEPVHEYEYNQ 280
PKNH    EMP------EYADHYHYGGN------KED-----------PDDMDYENGEEYDAQKYQDD 225
PVP01   VTP------EYADHYHYGGH------EED-----------PEDMDYENGEEYDAQGDPDD 283


PF3D7   ------------------------------------------------------------ 163
PmUG01  VYGSDHEDETNHETEFKQEETHNYEYEAKQED--EHKEHYEHSHTNEPEKEVAHSYEAEH 476
PocGH01 G------EEPVHEYEYNQGEEPVHEYEHNQEGESDN--------A------YEL-YHGGD 319
PKNH    H------YHHDEDYEYEEQDHANHDNHNDHDDHNDHDDHNDHDDH------NDHDDHNDH 273
PVP01   H---------DEHYEFD------------------------------------------- 291
```

```
PF3D7   ----------------------EQNTNTNKTLEYISDNLNDLINFKNIHLNNNSTGDFII 201
PmUG01  KKEDKPMQENEENYQYGDDQEYDEEEFKNKNVEYIQKKLKNLLAMKSIKLNNNSNGEFKV 536
PocGH01 I--------TEEYYDNEGH---NEENYKNKNIKYVGKKLKNLLAMKNIKLNNNSTGEFKV 368
PKNH    N--------DHNDHDDHNDHNDHDDEEEKQKIKYVGKKLKNLLAMKNIKLNNNSTGEFKV 325
PVP01   ---------EHDEHDEHGEHDEPYHEEDKKKIKYVGKKLKNLLAMKNIKLNNNSTGEFKV 342
                                .   ::.::*: .:*::*: :*.*:*****.*:* :

PF3D7   KLYTNYVNYIN-PYQTNPLPNTPHYYEHKNFHTKEHYI-------YDEEIVNPNMDNINT 253
PmUG01  NFYTNYVNYINQPPSNS-GSNHQEKYEYTEINQESKKKKKKS-----------------D 578
PocGH01 NFYTNYVNYIS-PYSINPFPSSQDMHKDNKVYEEGRNGKEEKYDEYSGGVSDGDK--DDS 425
PKNH    NFYTNYMNYINTPYVEPPFPFHKDIYEYAEVYSGDKIYPKD------------------- 366
PVP01   NFYTNYVNYINTPYGAPLLPFHKDSYEYAEVYSGDKLHPKK------------------- 383
        ::****:***. *          . ::  :.    :

PF3D7   HTEEDNVYYV-------SATKGNQKEETEK-KENHENNNAINPKYMN------------- 291
PmUG01  KMDYNSYFVKEVPTVVGEDEG--------EGDDIVNTNDIKQVNENLYNTNKAYDHMYNK 630
PocGH01 SGSHGGYYAGK-------------EEGETEDGYNKYRNAIRNIYEKMNNQSE-EEHEYEE 471
PKNH    HKDDNIYYGGENELIPQHVKGEMQKEALQEGSYHGYKSTMKGMYENIKKRSG-KK----- 420
PVP01   HGDEQMYYVGEKELIPVHGKGDMQK----EGPYDVYKGAMKGIYENIKKKAA-KK----- 433
          .    :                             . :.    :

PF3D7   --------------YETYYKKIFNAIFEQIDKLNKTLFEIKNKNNSETNEN--------- 328
PmUG01  K------IEGMEEKANKYYPYDDSS-----------EYA--------DNARYLKKGN--- 662
PocGH01 NAGEEQGKEGNKEWERDWYKDENSE-----------G------ERCKRDEHFV------- 507
PKNH    ------------ECKSGWCGKKMAG-----------KYQMMHDNNCNNDENNDG------ 451
PVP01   ------------GGKNGWWGKKKAG-----------KYQIKEDSNGDDDDDEDGDGDGDD 470
                         :                              :

PF3D7   ------------------------------------ISESNSGNPELNNENSYSVKLSSS 352
PmUG01  ----------------ELNKEGNNYYYYDDGKTQHRRSN----EYEQDRGNINGMKMSNK 702
PocGH01 ---------------EEETEYAD-SYFDGEDDV-NRKYTEEEQTEYSQKDDVEGMQLGNK 550
PKNH    ---------NDHDDHDEDDQLQNELLHQGHGTKKKSKCAHN--NKKLHGQNVDDKEITDK 500
PVP01   DDDDDNGDDDDDDEDPDEDDQLQNEPLHKGHGPNKKPKYGHK--KKKIHGENVDDEETTDK 528
                                                       .  :  . :  ..

PF3D7   SPNSTNKESLIFPYTYYNPYYMFRLTNNFKENDEGLK-------NENNI----------N 395
PmUG01  KPGKSEAKASHFPYTYYNPYYMYSLNSTGSPKYNSNSKYNNGYTNGHNNEYNYEYSNKYN 762
PocGH01 -SPTGEKKGLNFSYTFYNPYYMYRLGSKIPDNEKINK-NDKSYHIVKK------------ 596
PKNH    APAEKEKKGIDFSYTYHNPYYMFKLGSSMPTGK-------------GGH---------LK 538
PVP01   APSEKGKKGIDFAYTYYNPYYMFKLGSNMPTGKKAQP-SGKGAPAKGGL---------LK 578
               :.  * **::*****: * ..

PF3D7   -------------------NN--EDNQNDNMN---------------------------- 406
PmUG01  DKYNDKYNNKYSNKYSNKYNNEYNNEYNNEYNNEYNNKYNNEYNNKYNNKYNNEYNNKYN 822
PocGH01 -----------------------QEKLVGE------EKWK-------------------- 607
PKNH    ---------------GGKDDKEEEEEEVDD------------------------------ 553
PVP01   ---------------GGKGHDEEEEEVADE------EEEEEE------------------ 599
                               :::  .:

PF3D7   -------------------------------------------------IVLGKIHNIL 416
PmUG01  NEYNNKYNNKYNNKYSDKYSDRYKKYNNNYYNNYNKKQNKKSLYDFMLQKKALEKE-HML 881
PocGH01 --------------------D--------------------------------------- 608
PKNH    ------------------------------------------------------------ 553
PVP01   --------------EAEEVADAE------------------DVADEEDAEEVADED-ADV 626
```

```
PF3D7   KDFNINENIMTNKMSAPLIMTIILNFFKKYMAENKFNLPMSSEVENKINKSNNKALLQQS 476
PmUG01  KNNYENQESNTKKNKIGLFLDTLTE-LARYIESTINKNKINASKMKNNQLGDNKHDAIQQ 940
PocGH01 -STSIDYENITNKNSVEYFFNTLLE-LADYVAKNMNKSNLSGNGKKK-KHLGDQHVLLQN 665
PKNH    --ENGMEKNIEGESGANTFVNTLLE-LEGYVQPKESEH--------S-ELKEDKNTLGQS 601
PVP01   TDGDDAEKQHAKKSAANLFVNTLLE-LAGYLEPSESKK--------S-ELKEDKNSLGQA 676
               :    :     :.  : : :  *:  .  :         . :   ::    *

PF3D7   NKDTPIHKKKEIRNKKIQTKVDVIDEKTKKKIANTIYVNVGQSGINGFFNFFDFREKSID 536
PmUG01  SKKRPIHKRKEMKSRKMKTKTDLVDEKEKEKIQDTMFVKIGQNGTIGLLNFFDFREGLLK 1000
PocGH01 GKNRPIYKKKEMKSKKMKTKKDEIDENMVNKIKDTMYFKVGQNGTNNFLNFFDFREDTLK 725
PKNH    NKNRPIYKKKEMKSRKMKTKKDIPDEKITEKVKDTMYVKVGQNGTNGFLNFFDFREHSLK 661
PVP01   NKSRPIYKKKEMKSRKMKTKKDIVDEKTTEKIKDTMYVKVGQNGTNGFLNFFDFREYAVK 736
        .*. **:*:**::.:*::** *  **:  :*: :*::.::**.*  .::*******  :.

PF3D7   SNIFDLLHVMEDMKIFDIFQTIIFIQKFTENVCASYCMNITDVLELSHYDMIFYDKMVFH 596
PmUG01  NNFKDLIYVMEYLNVFNITETIMFIQKFTESICASYCMGITNVLELSNNDMLLYEKMRLI 1060
PocGH01 KNFTDLLLIMEQLKVFTISETIMFIQKFTETICASYCMGITDVLELSNNDMLLYEKMSIH 785
PKNH    KNFEDLLKVMEFLKVFNITETIIFIEKFTQSVCASYCMGITDVLELVNNDMLLYEKMSFH 721
PVP01   ENFKDLLKVMETLKVFNITETIIFIQKFTESVCASYCMGITDVLELVNNDMLLYEKMSFH 796
        .*: **: :** :::* * :**:**:***:.:******.**:**** : **::*:** :

PF3D7   FSKDGMMIKT-DKKYLYNLKEFENILNLLNINANTIALNCTCKFYVDVNYTYSEQYKMHL 655
PmUG01  FNNQGMTVVTKNSEYEFNDAEFEKFLLLLNLNKYTIPLNCPCKFYTNNIISYYIQYNSGL 1120
PocGH01 FDSKGMTVILENTEHHFSDTEFERILNLLNINKDTIPLTCPCKFYTNSIISYCKQYKSSL 845
PKNH    FHKNGMIVTT-NSNYEFNGIEFESLLDLLNINKETIPLICPCKSYTNNIISYCQQYKYNM 780
PVP01   FRKNGMTVTT-NSNYEFNGINFESLLALLNINKETIPLTCPCKSYTNNIISYCKQYKSNL 855
        * ..** :   :.:: :.  :** :* ***:*  ** * * ** *.:   :*  **:  :

PF3D7   KGYLHKMNEFDYINNFSASYLLNQLIIFQDKFNYIKMNGKLPIDDPKN---IYNMNNVHD 712
PmUG01  KGNFYQLKKSQFTEKFSPSYLLDKLIVLQDKLNYIRKHGKLEVNKRAVKNEDDDDDDFHE 1180
PocGH01 KGYFEQSKKAEYINKFSISYILQQLENLQEKLYYIKKNGKLECKQSSK---CYDGNDLYN 902
PKNH    KGYFTQSNNSEYLEKFDAYYLLEDLILLEEKLNDIKKNGKISSDKFVN---ILDSKDLYT 837
PVP01   KGYFTQSKNSEYLEKFTPYYLLEQLILLEDKLNYIKKNGKISSDTSVK---NLESKDLHT 912
        ** : : :: :: ::*   *:*:.*  :::*:  *: :**:  .         : .:.:

PF3D7   TAYYHNSRYFPTKDMPSLE---------------------------------------DN 733
PmUG01  NVYYHKQRYIPTIRTLNVENNSNT---------------------------------TDN 1207
PocGH01 TAYYHNERYFPTITTLNVSDGCSNGND-GSDGSDRSDGSGGSGGS-DGSDGSGDEGNQVK 960
PKNH    TAYYHNSRYFPPLRTCSASTTAPVINVSDKSAPNLASGFGEKVVDEIGLSKHGSNKAGVA 897
PVP01   TAYYHNNRYFPPLKASSASSTAPVSAVGGKSGLNRVGGFGGNGVDGNGVSSHGSNHGGGE 972
        ..***:.**:*     . .

PF3D7   FYEHLKYPDINTIHIYYNASPVKLNEVNDLKTIIIDEIKSKIFYINSYRVGDQFFPTYSN 793
PmUG01  TVNNFTYPDMDLLIVYYNSPLVNFKNLTDVKNILIEEVNSKIFYINSFRIGNQFFPTYSN 1267
PocGH01 TEVHLKYPDVETINVYYNASPVNLKNVNDVKNVLIEEVKSKIFYINSYRIGNQFFHTYSN 1020
PKNH    TGDKLTYPDVDALNIYYNASPVNLKNIQDVKSVLIDEVKSKIFYINSYRIGNQFFPTYSN 957
PVP01   LADELKYPDVDTLNIYYNASPVNLKSIHDVKNVLIDEVKSKIFYINSYRIGNQFFPTYSN 1032
           .:.***:: : :***:  *:::.: *:*.::*:*::********:*:*:*** ****

PF3D7   LGKDDHDLEHSAKNFYNISNENGDNTFNNNNNNMDNKKRMYNYNKHKDNDSRYTDNSNKN 853
PmUG01  LGKDDHDLEILEEVNASKINAPSRN----------------------------------- 1292
PocGH01 FGKDDHDLEIMEAVNTSKSKGENNS----------------------------------- 1045
PKNH    QGKDDHDLEIMQSDNSSKMNVEDKT----------------------------------- 982
PVP01   LGKDDHDLEILESANSSKLKVEGRT----------------------------------- 1057
         ********       .  :  . .
```

```
PF3D7   RDNSNKNRDNYNRNKDKNNTNRDNYNRYKDNNYYYNNSDNNNYNERKRYIRKKTYNKLSY 913
PmUG01  ------SGKSTSKST------------------------------TSNQNNGKHVSHLGC 1316
PocGH01 ------VRQEYYRNGKKEG-NRRG---------------EGKYVGGKGGEKSRVNSELAY 1083
PKNH    ------N---------------------------------------------KRIPKLGH 991
PVP01   ------S---------------------------------------------KRSPKLGH 1066
                                                            :   .*.

PF3D7   FNLPSLKSIYNNKIKGNSEEF---------------------------SFDNELPEQTES 946
PmUG01  FTLPNVKSLDKHNNKQNEDNF--------------------------LSSPIKVQGDCEW 1350
PocGH01 FASPTVDELLKQSKGENSGHSADSADASSIANT------ANAVDVEGDDQSDSARDSGDW 1137
PKNH    FVVPKLESVQKHSKEENKNAC---------IGGCSDDSSDDCSEDDESHVKHGIVGNSDL 1042
PVP01   FVVPKLESVEKHSTGGSGGSDGSDEDSDETTDAGSDGGSDGGSVGGGALAKHKIMGNSDW 1126
        *  *.:..: ::.   .                                       . :

PF3D7   FPLNKPQDHEAFYNLKKHHTNVYEPNDEEKQNEQKL--KDQIKITSDILYKDIEENKNTE 1004
PmUG01  VPLKSPQNYEDFYNEKKRNTGLFEKSEESN-NENKL--SDDLNLKYNFFENGDIDNKKEE 1407
PocGH01 VPLKSPKNHEDFYNERKRYTNLFEDKTEKK-KKNVV--EDRLNLKYDFLEKEDKE----- 1189
PKNH    IPLKSPPNHEDFYNAKKLQMNLFDHGGNKL-NEKEMEEKDGFILKFTSLEKGARANKAPE 1101
PVP01   IPLKSPPNHEDFYNAKKRHTNLLDHEGKQL-CEKKMDDSDCVSLKCAFLEKDARAKESGE 1185
        .**:.* ::* *** :*   .: :   :.   :: :  .* . :.   : :

PF3D7   DVLLIETITINNGTTSNTIENNKDSNKEAENSNTEQNDNNNNDNNNNINNNNNNNDNKEE 1064
PmUG01  DLYAK-NIHANDTSETDSLPLNGKTK----------K---------------KYHSNKS- 1440
PocGH01 ----------------------------------------------------NYNTDDT- 1196
PKNH    TVEKNS-------DEGDNQKIDGGVTTG----GEDQA-----E-----------TENDE- 1133
PVP01   CDKEVVDKHTGDKEASDKQTGEGGET-D----GADQA-----E------SQNNVNESEE- 1228
                                                                ..

PF3D7   DMNENNNNSKVTGDSVENINEQTNNNQYPNTEYNTIQRSINAKYLIFFFKNLHVWKTDLF 1124
PmUG01  -----------------NI-SSNSNSNNIGNNDSNVKKSIKAKYLIYFFKNIHVWKTQVY 1482
PocGH01 ----------------NLINSVKLKSRMRGTNAEKKKKVINAKYLIYFFKNIHVWKTSVY 1240
PKNH    ----------------HSVDSLKLLNKMEKTYLHKKKKTISAKYLIYFFKNVHVWKTGEY 1177
PVP01   ----------------HSAESLKLQSKMGEPLSCKKKKAISAKYLIYFFKNVHVWKTGEY 1272
                            . .  ..       . :: *.*****:****:*****  :

PF3D7   CQNINYMNNYLNSIQYNKTLTFDINYDTNAVTIYFTDNITYTVKVNLEYLVFLLEKISLI 1184
PmUG01  CQNMNYINNLLENINYDEDIIFQENLEEDIVYVYFTSKLGVKYDIDVNYFIFLLQKISLI 1542
PocGH01 CQNMNYIDNLLKNINYNEDIVFQESVENGTVSLLFSSNLKDIYKIDMDYFIFLLQKISLI 1300
PKNH    CQNMNYIDKFLKSINYKEEITFQEHLDQDSVVLQFTSSLKNIYKVDMDYFIFLLQKISLI 1237
PVP01   CQNVNYIDNWLKKINYNEEIIFQEQLDEDSVVLYFSSNLKDKYKLDMEYFIFLLQKISLI 1332
        ***:**::: *:.*:*.: : *:   : . * : *:..:    .::::*::***:*****

PF3D7   TFVEDLCSLFDTDKKRNYKNLTEFLENTERINTFVRNHMMLSNEQFINKNKYAKELAEIS 1244
PmUG01  MYVEDLCGIFQLDDVKQNQKLDRHLENTTNTQNFIEKHIIFFYEQYSNKNKYAKQLSNIS 1602
PocGH01 MYVEDLCGIFQLDEVKKNKKIDKYLEKKGNIHHFLEKHIIFSHEQYIKKNKYARKLSDVS 1360
PKNH    MYVEDLCEIFQIEEMKQNKKIDKYIENEANIVNFIEKHLMFSHEQYSKKNKYAKELSIIS 1297
PVP01   MYVEDLCGIFQIDEMKQNKKIDKHIENPANIHNFMEKHILFSHEQYSKKNKYAKELSIIS 1392
         :***** :*: :. :: ::: ..:*:  .   *:.:*:::  **: :*****::*: :*

PF3D7   TSNLFYPKKDIILRSTPYNNIILDEKDIYQTIFIYMDDMLTEKMVNDTWITPYAFVVYSK 1304
PmUG01  TSTFFSSKKDIILNTESYNNIIFNEKDIYDVMFIYMEDVLTEKMVIDTWLTPYGFMLYKP 1662
PocGH01 TSNFFSSKKDIIFNPEPYNNVIFNEKEIYESLHTYMEDVLTEKMLNETWLTPYGFMLYSP 1420
PKNH    TSNLFSSKKDIILNSQPYNNIVFNEKEIYESLFIYMEDVLTERMMNATWLTPYGFILYKQ 1357
PVP01   TSNFFSSKKDIILNSQPYNNIVFNEKEIYESLYVYMEDVLTERMINETWLTPYGFILCKP 1452
        **.:*  *****:.   ***::::**:**: :. **:*:***:*:  **:***.*:: .
```

```
PF3D7   SKKD--MQGNNIKIEQNKNITKYSRSAIDKYVHYEYKRISENLNRFFMESNSNAPQFNEN 1362
PmUG01  NNDSGNNDNIKLSISKNENITKHSRNSIDKYLFYEYKKISNNILQYYDDLNSKIPQFKDN 1722
PocGH01 NK-A--NSKYKLKISQNVHITKYSRNAIDKYIYYEYKKISNNIIQYYEELNPKIHEYSDD 1477
PKNH    PSKD--LNSYKLKIAQNEFITKFSRSAIDKYMYYEYRKISNNIVQHHVELSPKLGENLAE 1415
PVP01   TLSD--ANNYKLKISQNEFITKYSRSAIDKYMYYEYRKIGNNIVQHHLDLSPKLSEHLAE 1510
               .  ::.* :*  ***.**.:****:.***::*.:*: :.. : . :  :   :

PF3D7   YKEYTIIIYNDNPSMPNIVLTTTINVFNVSFLQSIMEMLLNIKANQQFFSYKGKFIPINA 1422
PmUG01  LKEYKLVIYNDNPSMTNIVLTTTINVLNVALLQSILEVVLEIKANQQFFSYNGKFISLNT 1782
PocGH01 LKEYKLVIYNDNPSMTNIIIRTTVNVLNIAFLQSLLEVILDIRADQQFFSFKGKFLPVNA 1537
PKNH    PKEYKLIIYNDNPSMTNIIITTSINVLNISFLHSLLEIILDIRATQQFFSYKGRFIPINA 1475
PVP01   LKEYRLIIYNDNPSMTNIIITTTVNVLNIAFLQSLLEVILDIRATQQFFSYKGRFIPINA 1570
         *** ::******** **:: *::**:*:::*:*::*::*:*:* *****::*:*: :*:

PF3D7   FITLENKINYIFFNYIPLENYVNNGDALDFRNP      1455
PmUG01  FIILDEGINYLFFNYIPNENHPALSNTGYA---      1812
PocGH01 FLILDEGINYLFFNYVPNENHINFTCQV-----      1565
PKNH    FIILDEGVNYLFFNYAPNENHINEEA-------      1501
PVP01   FIILDEGVNYLFFNYVPNENHINYAA-------      1596
        *: *:: :**:**** * **:
```

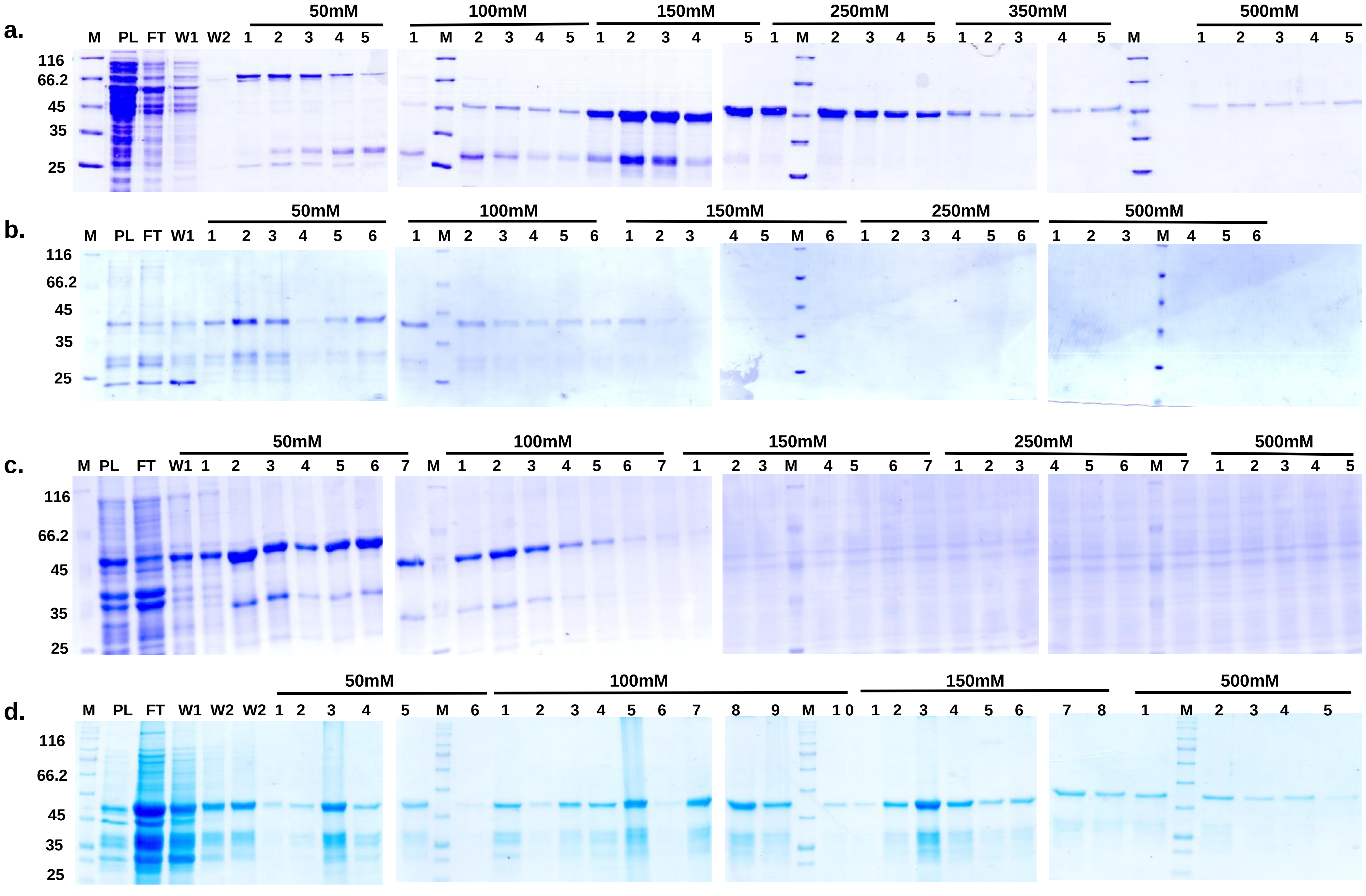

Vanndita Bahl et al (2021)
Supplementary Figure 2

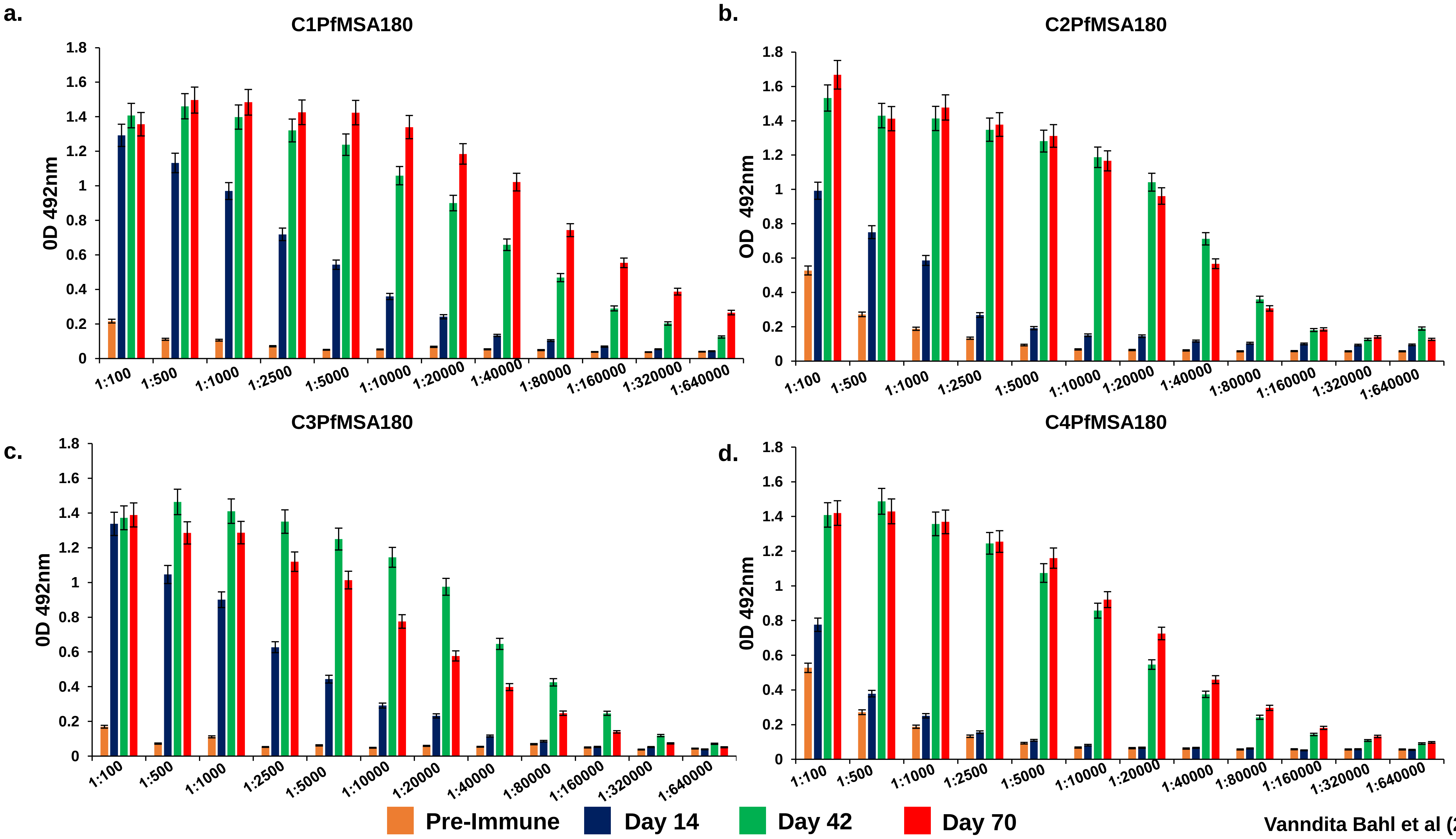

Vanndita Bahl et al (2021)
Supplementary Figure 3

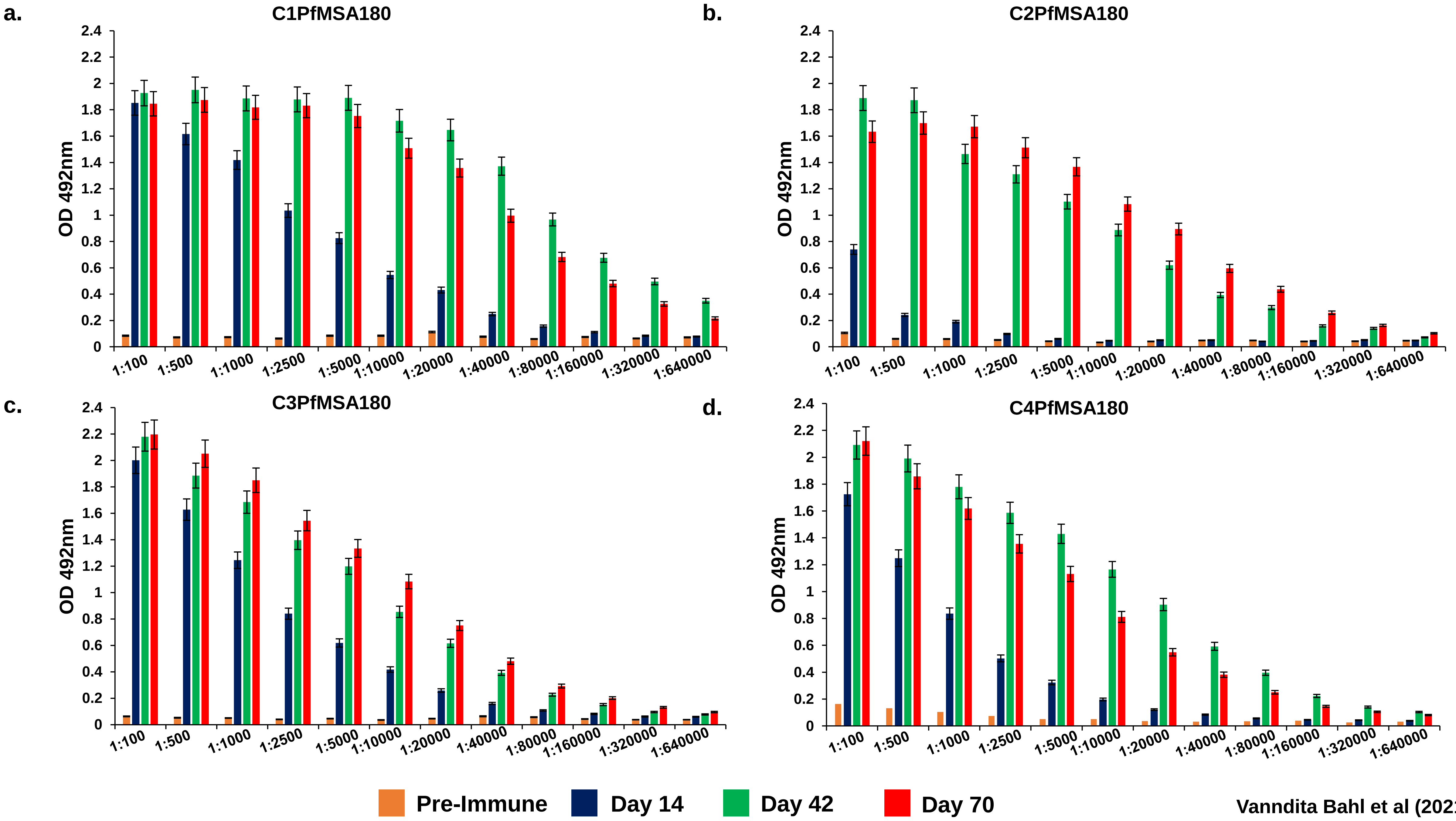

Vanndita Bahl et al (2021)
Supplementary Figure 4

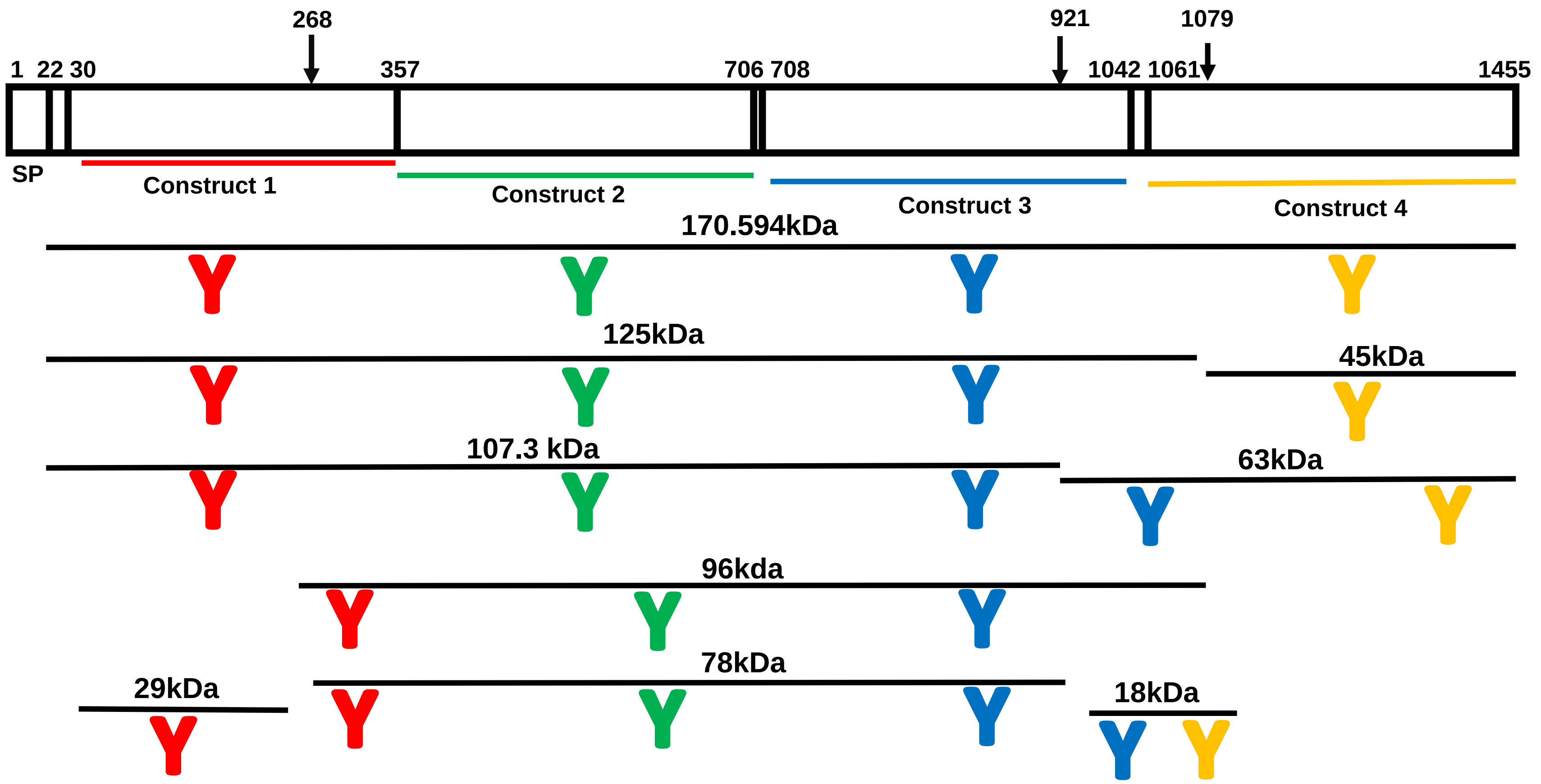

Vanndita Bahl et al (2021)
Supplementary Figure 5

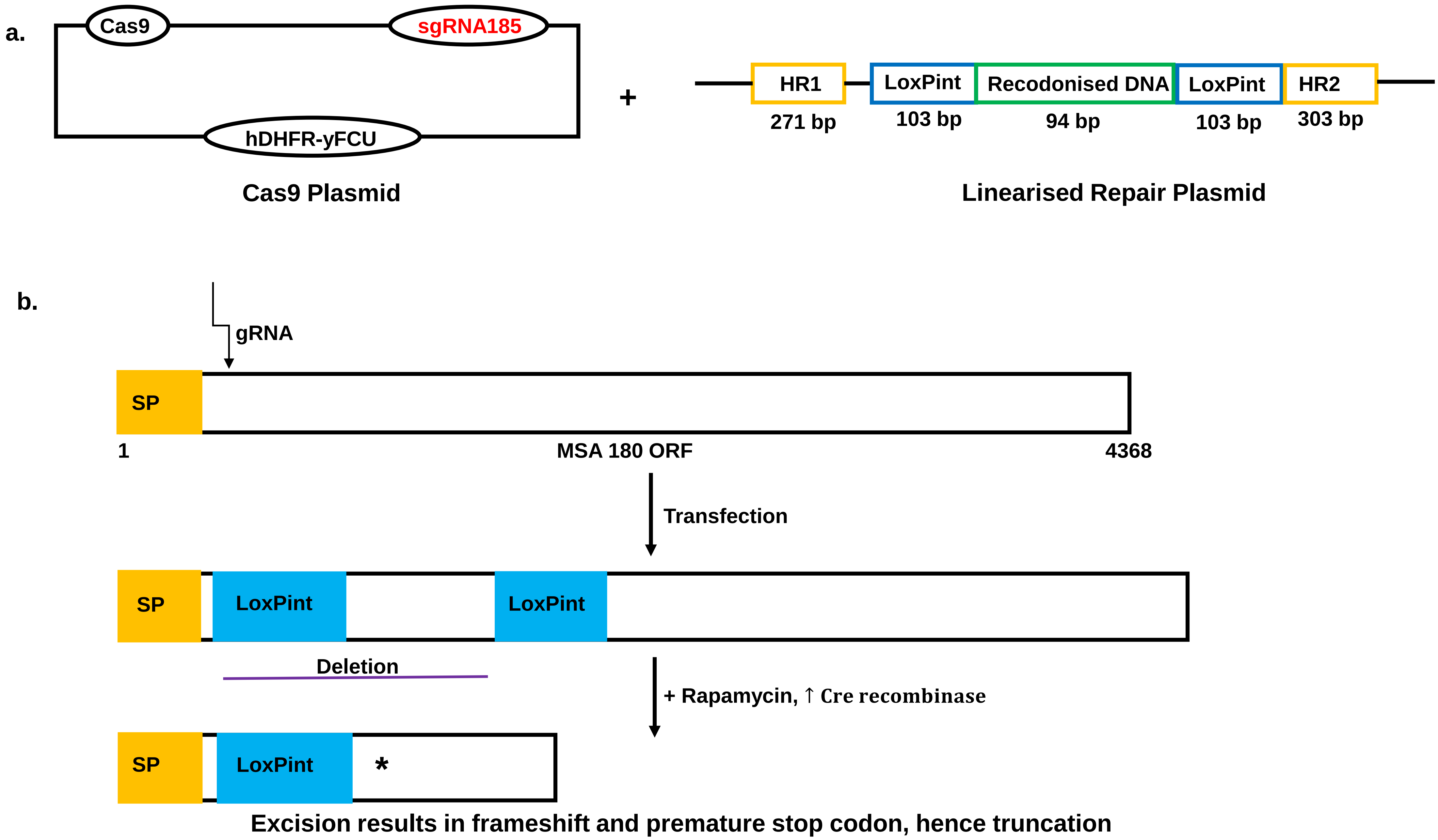

Vanndita Bahl et al (2021)
Supplementary Figure 6

a. **SEQUENCE BEFORE EXCISION**

ATGAATCGAATATTTTATTTTTGTTTGTTTACTATTTTGTTTTGGTTATCTCTTGTATCTGGTGAAAATGTTAATAATAAAAACTGTAATGAGAAAAATAGGAAAGCTATTTTAT

TAGCTTTATTAAAAAATTCATTAGTAGATAATAAGGATTATAACAATAGTGAAGAATTAAAGTATGCTCTTGAGCACATACA gtaaataaaaaaaataatatacaATAACTTCGTATAG

CATACATTATACGAAGTTATtatatatgtatatatatatatatttatatattttatattcttttag GAACAGTGAGCTATACCCTAAAGACTCTAAGAAGTTCGATAAGTTTATAGACGAGTTCTTCTC

TTACTACAACATACACGTTAACTTCACAGAC gtaaataaaaaaaataatatacaATAACTTCGTATAGCATACATTATACGAAGTTATtatatatgtatatatatatatatttatatattttatattcttttag

GAAGAAAAAAGAATATTACATATATCAGGTGTCTTCAAAGAATTTTATGTAGATGTAGATAATTTAAATAAAGATGAAATGAAAGAATATTTTAAGAAAAATTATGAAAAAGG

b. **SEQUENCE AFTER EXCISION**

ATGAATCGAATATTTTATTTTTGTTTGTTTACTATTTTGTTTTGGTTATCTCTTGTATCTGGTGAAAATGTTAATAATAAAAACTGTAATGAGAAAAATAGGAAAGCTATTTTAT

TAGCTTTATTAAAAAATTCATTAGTAGATAATAAGGATTATAACAATAGTGAAGAATTAAAGTATGCTCTTGAGCACATACA gtaaataaaaaaaataatatacaATAACTTCGTATAGC

ATACATTATACGAAGTTATtatatatgtatatatatatatatttatatattttatattcttttag GAAGAAAAAAGAATATTACATATATCAGGTGTCTTCAAAGAATTTTATGTAGATGTAGATAATTT

AAATAAAGATGAAATGAAAGAATATTTTAAGAAAAATTATGAAAAAGG

c. **PREMATURE STOP CODON AFTER EXCISION**

ATG AAT CGA ATA TTT TAT TTT TGT TTG TTT ACT ATT TTG TTT TGG TTA TCT CTT GTA TCT GGT GAA AAT GTT AAT AAT AAA AAC TGT AAT GAG AAA AAT AGG

AAA GCT ATT TTA TTA GCT TTA TTA AAA AAT TCA TTA GTA GAT AAT AAG GAT TAT AAC AAT AGT GAA GAA TTA AAG TAT GCT CTT GAG CAC ATA CAG AAG

AAA AAA GAA TAT TAC ATA TAT CAG GTG TCT TCA AAG AAT TTT ATG TAG ATG TAG ATA ATT TAA ATA AAG ATG AAA TGA AAG AAT ATT TTA AGA AAA ATT ATG

AAA AAG G

LoxPint sequence (ATAACT------TTAT: LoxP Sequence)

MSA180 Recodonised DNA

Vanndita Bahl et al (2021)
Supplementary Figure 7

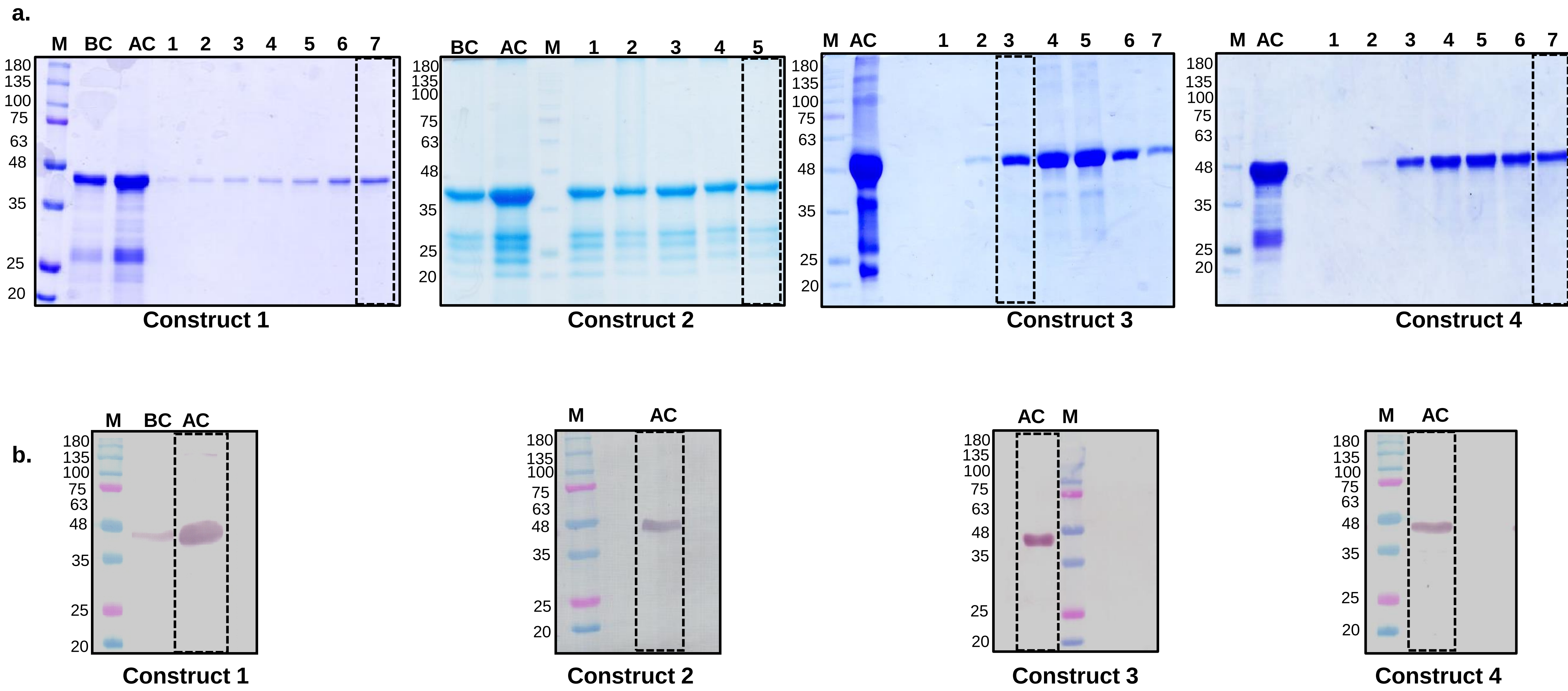

Vanndita Bahl et al (2021)
Supplementary Figure 8

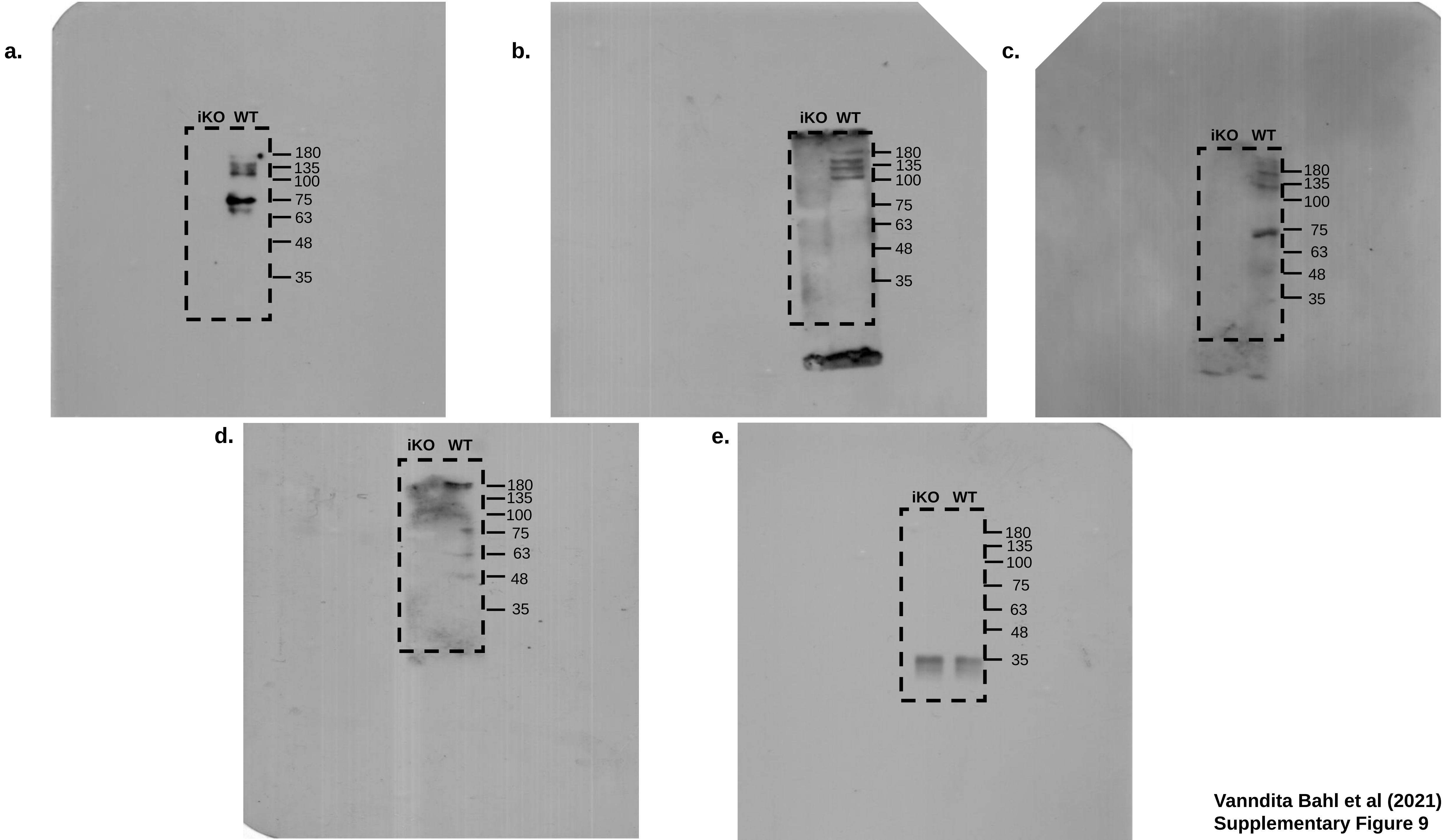

Vanndita Bahl et al (2021)
Supplementary Figure 9

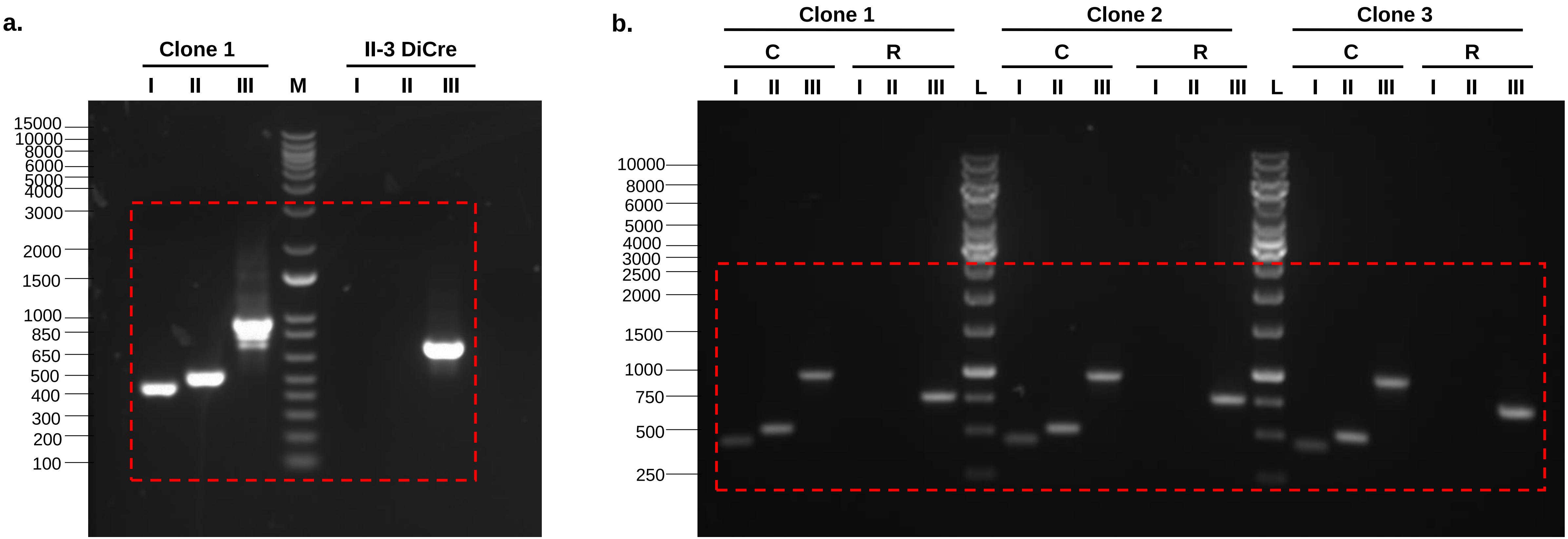

Vanndita Bahl et al (2021)
Supplementary Figure 10
